# *In vivo* covalent cross-linking of photon-converted rare-earth nanostructures for tumour localization and theranostics

**DOI:** 10.1038/ncomms10432

**Published:** 2016-01-20

**Authors:** Xiangzhao Ai, Chris Jun Hui Ho, Junxin Aw, Amalina Binte Ebrahim Attia, Jing Mu, Yu Wang, Xiaoyong Wang, Yong Wang, Xiaogang Liu, Huabing Chen, Mingyuan Gao, Xiaoyuan Chen, Edwin K.L. Yeow, Gang Liu, Malini Olivo, Bengang Xing

**Affiliations:** 1 Division of Chemistry and Biological Chemistry, School of Physical and Mathematical Sciences, Nanyang Technological University, 637371 Singapore, Singapore; 2 Singapore Bioimaging Consortium, Agency for Science Technology and Research (A*STAR), 138667 Singapore, Singapore; 3 Department of Chemistry, National University of Singapore, 117543 Singapore, Singapore; 4 State Key Laboratory of Molecular Vaccinology and Molecular Diagnostics, Center for Molecular Imaging and Translational Medicine, School of Public Health, Xiamen University, 361102 Xiamen, China; 5 School of Radiation Medicine and Protection, Soochow University, 215123 Suzhou, China; 6 Institute of Materials Research and Engineering, A*STAR (Agency for Science, Technology and Research), 117602 Singapore, Singapore

## Abstract

The development of precision nanomedicines to direct nanostructure-based reagents into tumour-targeted areas remains a critical challenge in clinics. Chemical reaction-mediated localization in response to tumour environmental perturbations offers promising opportunities for rational design of effective nano-theranostics. Here, we present a unique microenvironment-sensitive strategy for localization of peptide-premodified upconversion nanocrystals (UCNs) within tumour areas. Upon tumour-specific cathepsin protease reactions, the cleavage of peptides induces covalent cross-linking between the exposed cysteine and 2-cyanobenzothiazole on neighbouring particles, thus triggering the accumulation of UCNs into tumour site. Such enzyme-triggered cross-linking of UCNs leads to enhanced upconversion emission upon 808 nm laser irradiation, and in turn amplifies the singlet oxygen generation from the photosensitizers attached on UCNs. Importantly, this design enables remarkable tumour inhibition through either intratumoral UCNs injection or intravenous injection of nanoparticles modified with the targeting ligand. Our strategy may provide a multimodality solution for effective molecular sensing and site-specific tumour treatment.

## Abstract (web summary)

Directing nanomedicines to desired locations - such as tumour sites - is difficult to achieve selectively. Here, the authors develop a method to covalently crosslink peptide-modified upconversion nanocrystals into tumour sites for photodynamic therapy and show *in vivo* tumour inhibition in mice.

## Graphical abstract

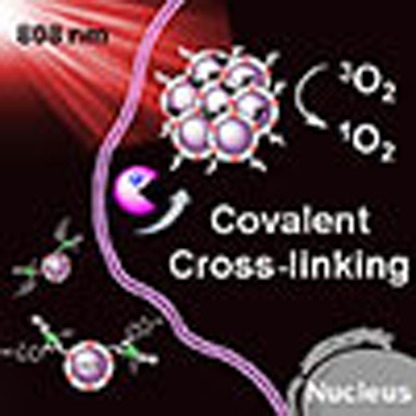

Currently, therapeutic and diagnostic techniques based on supramolecular assemblies and functional nanomaterials have been extensively recognized as promising nanomedicine platforms for the battle against many urgent health concerns including cancer, cardiovascular and neurodegenerative diseases as well as other life-threatening illnesses^1^,^2^,^3^. The remarkable biomedical application of nanomaterials could be mainly attributed to their unique photo-physical properties, high surface area and multivalent binding ability^4^,^5^. Despite the leap forward in the continuous breakthroughs in biomedical research, critical challenge still remains in designing targeted nanoplatforms that are capable of selectively localizing at the specific diseases—in particular—tumour sites for early-stage diagnosis and effective treatment^6^,^7^,^8^. One emerging strategy to achieve high targeting selectivity is to conjugate the nanomaterials with affinity ligands including small organic moieties or bioactive molecules that can bind to receptors in the tumour cells^9^,^10^,^11^,^12^. However, varying expression levels of the receptors, complex and dynamic physiological cell environments may potentially pose the issue of nonspecific recognition for this ligand-mediated tumour affinity. Therefore, more specific targeting approaches are demanded that do not solely rely on receptors to differentiate tumour and normal cells^11^,^12^. Indeed, some bioorthogonal reactions provide feasibility to locate functional nanostructures into tumour cells mostly through their electrostatic or covalent binding to biomolecules in living system^13^,^14^,^15^,^16^,^17^,^18^. Nevertheless, the effective bioorthogonal functionalities that can selectively respond to the dynamic processes of native environment are still ongoing challenges for *in vivo* applications^18^,^19^,^20^. Hence, different approaches that enable sensitive recognition of dynamic tumour microenvironment, and more importantly, can further trigger the tumour-specific localization of theranostic nanomaterials *in vivo* are highly desirable, and extensive studies still need to be further investigated.

Recently, rare-earth doped upconversion nanocrystals (UCNs) have been widely demonstrated for use in biomedical applications. In general, UCN particles offer deep tissue penetration capability for enhanced bioimaging and better tumour treatment arising from their unique non-linear photon upconverting process upon light irradiation at near-infrared (NIR) window^21^,^22^,^23^,^24^,^25^,^26^,^27^,^28^,^29^. As with the majority of nanomaterials for theranostic tumour studies, the effective targeting of upconversion materials mainly relies on receptor-mediated interactions, and the specific cellular localization of UCN nanostructures at the tumour site upon the sensitive response to microenvironment stimulation have not been fully solved^30^,^31^,^32^,^33^,^34^. Moreover, despite the great potential of UCNs in meeting biomedical demands *in vitro* and *in vivo*, most of the conventionally used UCNs employ long-wavelength laser irradiation at 980 nm. This often leads to an inevitable overheating effect because of significant water absorption^35^,^36^. To this end, the development of new strategies to selectively direct UCNs into targeted tumour areas, while notably minimizing undesired side effects is highly essential. Unfortunately, such relevant studies are still limited so far.

In this work, a tumour microenvironment-sensitive UCNs platform has been designed and prepared to perform *in vivo* covalent localization of particles at the tumour site. Different from the process involving nonspecific tumour targeting, such unique platform can respond to tumour-specific enzyme and undergo cross-linking reaction, which thus enables the selective tumour accumulation. More significantly, compared with the particles that cannot undergo cross-linking reaction, the enzyme-triggered covalent cross-linking of UCNs possess an enhanced light upconverting emission when illuminated at 808 nm. Such enhancement can effectively amplify the production of reactive singlet oxygen (for example, ^1^O_2_) from the photosensitizers loaded on the particle surface, which therefore represents promising nanomedicine for improved photodynamic tumour treatment (PDT) as well as non-invasive fluorescence and photoacoustic imaging *in vitro* and *in vivo*.

## Results

### Enzyme-triggered covalent cross-linking of UCN *in vitro*

**Figure 1:**
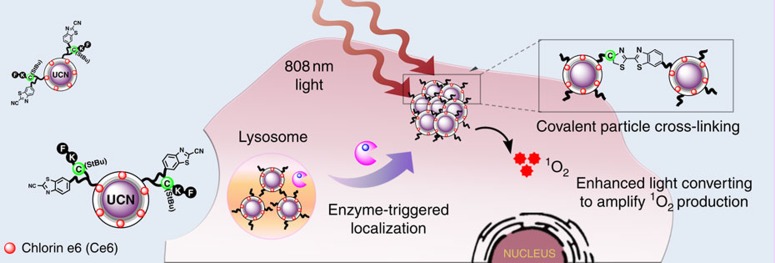
Illustration of the microenvironment-sensitive strategy for covalent cross-linking of peptide-premodified UCNs in tumour areas. Upon tumour-specific cathepsin B (CtsB) enzyme reactions, the peptide cleavage on the particle surface induces covalent cross-linking between the exposed free cysteine and 2-cyanobenzothiazole on neighbouring particles, which triggers the accumulation of UCNs into the tumour site. Such enzyme-triggered covalent cross-linking of UCNs leads to an enhanced upconversion emission after 808 nm laser irradiation, and in turn amplifies the singlet oxygen generation from the photosensitizers (for example, Ce6) attached on UCNs for enhanced PDT treatment *in vitro* and *in vivo*.

Figure 1 illustrates the rational design of our enzyme-responsive cross-linking of rare-earth UCNs (CRUN) for tumour localization and targeted therapeutics. Generally, the core-shell UCNs were synthesized according to an established thermal decomposition method^26^. In order to minimize undesired overheating effect typically associated with 980 nm laser irradiation, we constructed lanthanide platform doped with Nd^3+^ ions for improved pumping efficiency at 808 nm. These Nd^3+^-doped nanocrystals were further coated with polyacrylic acid (PAA) and polyethylenimine (PEI) to improve biocompatibility and to facilitate subsequent surface modification (Supplementary Fig. 1). Transmission electron microscopy and spectroscopic analysis revealed a cubic morphology of the particles with a narrow size distribution of 40±2 nm. Both core-shell UCNs and polymer-coated UCNs exhibited similar upconverted luminescences with visible emission at 545 and 655 nm upon excitation at 808 nm (Supplementary Fig. 2).

To ensure the theranostic efficacy, an effective photosensitizer, chlorin-e6 (Ce6), with favourable optical properties was chosen and coupled to the PEI/PAA@UCNs. In order to achieve selective tumour localization, an enzyme-responsive peptide, Ac-FKC(StBu)AC(SH)-CBT (Supplementary Fig. 3) containing a side-protected cysteine and 2-cyanobenzothiazole (CBT) was designed for specific reaction with cathepsin B (CtsB), one important lysosomal cysteine protease overexpressed in various malignant tumours to process intracellular protein degradation and regulate cancer pathology^37^. The selected sequence was further connected to Ce6-PEI/PAA@UCNs through the reaction of free thiol in terminal cysteine and short maleimide oligo (ethyl glycol; dPEG_2_) linker on the particle surface. The successful formation of peptide and Ce6-modified UCNs (CRUN) conjugate was confirmed by spectroscopic characterization (Supplementary Fig. 4). The optimal amount of peptide on UCNs was finally determined to be 11.4 nM mg^−1^, and there was 2.1% Ce6 found on the UCN surface (Supplementary Fig. 5). In principle, cellular uptake of the peptide and Ce6-conjugated UCNs may first reduce the disulfide bond of protected cysteine due to the reductive cellular environment. The subsequent lysosomal enzyme processing will cleave the peptide and expose a free 1,2-aminothiol group in cysteine, which easily undergoes condensation reaction with –CN moiety in CBT structure^13^,^18^, and thus triggers the localization of cross-linked UCNs at the tumour region (Supplementary Fig. 6). During the reaction, great care needs to be taken to avoid nonspecific cross-linking by providing optimal spacing in the enzyme-responsive peptide and the short PEG linker to control the distance between the peptide and particle surface.

**Figure 2:**
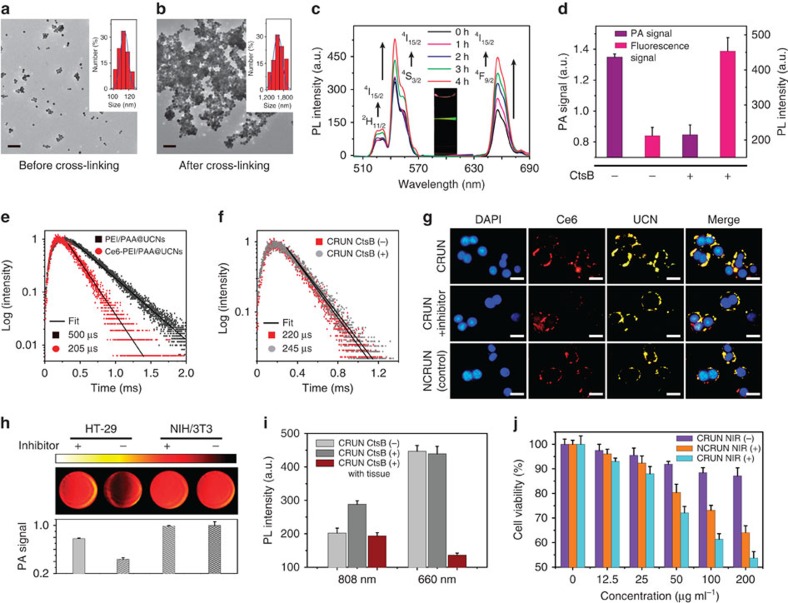
*In vitro* studies of tumour-specific enzyme (CtsB) triggered cross-linking of CRUN upon 808 nm illuminations. TEM and DLS analysis of covalent cross-linking of CRUN (1.5 mg ml^−1^) without (**a**) and with (**b**) CtsB (55 nM) at 4 h. The hydrodynamic diameters in DLS are about 110 nm (**a**) and 1,580 nm (**b**). Scale bar, 200 nm. (**c**) UCL of CRUN at different time intervals with CtsB treatment. The inset shows the luminescence photograph of CRUN incubate with CtsB for 4 h. (**d**) Photoacoustic (PA) imaging and UCL signal after 4 h incubation of CRUN with CtsB. (**e**) Luminescence decay curves of the emission at 655 nm of UCNs in the absence and presence of Ce6 excited by 808 nm laser. (**f**) Lifetime of CRUN in the absence and presence of CtsB treatment. (**g**) Confocal imaging of HT-29 cells incubated with CRUN, CRUN and inhibitor, and control NCRUN, respectively. Blue: DAPI (λ_ex_=350/50 nm, λ_em_=460/50 nm), red: Ce6 (λ_ex_=545/25 nm, λ_em_=610/75 nm), yellow: UCN (λ_ex_=980 nm, λ_em_=350–690 nm). Scale bar, 20 μm. (**h**) PA signals of HT-29 and NIH/3T3 living cells without (left) and with (right) CtsB inhibitor. The PA signals were shown with a laser excitation at 680 nm. (**i**) Quantification of singlet oxygen generation before and after enzyme-triggered CRUN or covered by 2-mm thick pork tissue when exposed to 808 or 660 nm laser irradiation for 1 h (0.4 W cm^−2^). (**j**) Cell viability of HT-29 at different concentrations of CRUN and NCRUN after NIR light treatment for 1 h. Data are mean±s.d. (*n*=3 technical replicates).

First, we examined the covalent cross-linking reactions of CRUN upon enzyme treatment in buffer through particle morphology and spectroscopic analysis. After incubation of CRUN (1.5 mg ml^−1^) with CtsB (55 nM), transmission electron microscopy and dynamic light scattering analysis indicated significant particle aggregation in solutions (Fig. 2a,b and Supplementary Fig. 7), while CRUN alone or the enzyme-treated CRUN together with CtsB inhibitor (antipain), remained unchanged over time. Furthermore, the absorption spectrum showed a new peak at 489 nm during the enzyme reaction, indicating the formation of firefly luciferin structure in solutions^13^. These results clearly suggest that the particles aggregation is mainly due to the specific CtsB enzyme-triggered cross-linking reaction (Supplementary Fig. 8). Compared with CRUN without CtsB treatment, the covalent cross-linking of CRUN could lead to an enhanced upconversion luminescence. There was up to 2.2-fold luminescence emission intensity enhancement observed at 655 nm after 4 h reaction (Fig. 2c, d and Supplementary Fig. 9). Similar enzyme-triggered CRUN cross-linking was also displayed by the luminescence colour change in the absence and presence of CtsB inhibitor (Supplementary Fig. 10), further confirming that it is the specific enzyme reaction that induces the enhanced luminescence from CRUN particles.

Considering deep tissue penetration capacity and great advantages of the photoacoustic imaging technique that integrates both functions of ultrasound and optical imaging, we assessed the difference in photoacoustic signal during the specific enzyme cross-linking reactions. Contrary to the increased luminescence observed, there was an obvious photoacoustic phantom signal decrease (∼40% in intensity) detected in solution after 4 h reaction (Fig. 2d). Generally, laser illuminations on enzyme-responsive UCN conjugates would excite the internal energy levels of lanthanide dopants. The excited states can relax to the ground state through both radiative and nonradiative processes. The enhanced radiative emission relaxation may correspondingly lead to a deactivation of the nonradiative relaxation in photoacoustic, therefore resulting in the decreased photoacoustic signals as observed in the experiments^38^,^39^,^40^,^41^. These results demonstrate that such inverse trend observed in photoacoustic may also be attributed to the specific enzyme-triggered cross-linking reaction.

Furthermore, we also investigated the underlying reason of the enzyme-responsive luminescence enhancement of CRUN by monitoring the decays of the luminescence lifetime. As shown in Fig. 2e, the presence of Ce6 on CRUN particles would lead to a sharp decay (from 500 to 205 μs) in lifetime at 655 nm, indicating the strong absorption of Ce6 at around 660 nm (ref. 35). Moreover, upon enzyme treatment, there was no significant lifetime change of lanthanide dopants in CRUN (Fig. 2f and Supplementary Fig. 11). In addition, we also examined the resonance light scattering properties of CRUN before and after CstB reactions (Supplementary Fig. 12). There was obvious scattering increment observed upon 808 nm laser excitation, mostly due to the covalent cross-linking of UCN conjugates triggered by enzyme reaction. The increase in scattering efficiency enhanced the amount of light absorption among the cross-linked particles, which could be the possible factor contributing to the increased upconversion luminescence^42^,^43^.

### Enzyme-triggered cross-linking of UCN in living cells

The intracellular uptake and enzyme-triggered cross-linking of UCNs in living cells were further investigated by fluorescence and photoacoustic imaging techniques. Typically, CtsB-overexpressing HT-29 tumour cell and CtsB-deficient NIH/3T3 cells were chosen as models to incubate with enzyme-responsive CRUN (50 μg ml^−1^) at 37 °C for 4 h. The different expression levels of CtsB were verified by western blotting and quantitative human CtsB ELISA kit in HT-29 (40.1±4.4 ng mg^−1^ protein) and NIH/3T3 (2.2±1.4 ng mg^−1^ protein) cells, respectively (Supplementary Fig. 13)^44^. As shown in Fig. 2g, the obvious yellow fluorescence contributed from light-upconversion emissions at 545 and 655 nm was clearly observed in the HT-29 cells, indicating the effective cellular internalization of CRUN. Moreover, compared with the cells incubated with CtsB inhibitor or control NCRUN conjugates (the control particles with surface modified by non-cross-linking Ac-FKC(StBu)AC sequence), HT-29 cells treated with enzyme-responsive CRUN exhibited an increased luminescence. In addition, there was no obvious fluorescence enhancement detected in CtsB-deficient NIH/3T3 cells after incubation (Supplementary Figs 14,15). These results clearly suggested that the overexpressed CtsB enzyme in HT-29 cell could induce covalent cross-linking of CRUN and such cross-linking leads to the desired particle localization in the tumour microenvironment.

Similar cellular studies based on photoacoustic imaging were also carried out to monitor CRUN localization in living cells. As shown in Fig. 2h, the enzyme-responsive CRUN, when incubated with HT-29 cells, exhibited less photoacoustic signal (∼56%) than that incubated with NIH/3T3 cells (CtsB-deficient). Moreover, processing of CRUN in HT-29 cells upon blocking the CtsB activity with inhibitors prevented the decrease in photoacoustic signal, corresponding to the specific enzyme reactivity. Moreover, incubation of CRUN with NIH/3T3 cells would not induce obvious photoacoustic signal change, further confirming that the enzyme processing can cause the localization of CRUN particles within HT-29 cells and the enhanced upconverting emissions resulted in the photoacoustic signal decrease. This was in agreement with the observation tested in buffer.

We also evaluated the capability of CRUN to generate cytotoxic singlet oxygen (^1^O_2_) through the standard singlet oxygen sensor green^45^. Basically, 808 nm laser irradiation of CRUN conjugates resulted in the converted emission 655 nm, corresponding to the absorption of photosensitizer Ce6 labelled on UCNs surface. Compared with CRUN alone or enzyme-treated CRUN with inhibitor, the CtsB enzyme-induced cross-linking can cause enhanced light converting emission, which thus amplifies the production of ^1^O_2_ from the activated photosensitizers after 1 h illumination (Fig. 2i and Supplementary Fig. 16). Importantly, a 2-mm thick pork tissue (adipose tissue) was also used to mimic clinical skin for further investigation of the different penetration of 808 nm (NIR) and 660 nm light irradiation. As shown in Fig. 2i, the presence of pork tissue significantly decreased the efficiency of ^1^O_2_ generation (2.9-fold decrease) with 660 nm irradiation, while there was only 0.6-fold decrease in ^1^O_2_ generation when illuminated at 808 nm. The higher singlet oxygen production with 808 nm excitation in the presence of pork tissue clearly indicated a better penetration depth as compared with irradiation at 660 nm, which makes UCN-based PDT suitable for effective tumour treatment in living system.

The potential photodynamic cytotoxicity of CRUN was further evaluated through an *in vitro* toxicology assay (TOX8). As shown in Fig. 2j, there was negligible cytotoxicity in the CRUN-incubated HT-29 cell without laser exposure at 808 nm, suggesting minimum toxicity caused by the particle itself. However, upon 808 nm laser illumination of CRUN (200 μg ml^−1^) incubated HT-29 cells, there was higher cytotoxicity (∼53% cell viability) observed in HT-29 cells than that of cells treated with the control NCRUN (∼64% cell viability). In addition, similar photodynamic inactivation was also studied in CRUN-incubated HT-29 cells but with CtsB inhibitor or in the negative control (CtsB-deficient NIH/3T3 cells). Compared with the laser-excited HT-29 cells with CRUN, there was no significant enhancement of cytotoxicity detected in these control experiments. Meanwhile, 808 nm laser irradiation alone did not induce obvious cytotoxicity in both HT-29 and NIH/3T3 cells (Supplementary Fig. 17), indicating that the covalent cross-linking of CRUN triggered by specific enzyme would increase the singlet oxygen generation, and thus induce the enhanced cell death in the targeted tumour cells with overexpression of the CstB enzyme. Moreover, the cytotoxicity of HT-29 cells incubated with CRUN was also evaluated upon 660 or 808 nm light irradiation. Although the photodynamic cytotoxicity was found to be higher at direct 660 nm excitation (∼86% in cytotoxcity) than that at 808 nm irradiation (∼39% in cytotoxcity) (Supplementary Fig. 18), the presence of pork tissue can significantly lower the cytotoxicity at 660 nm illumination (∼13%,∼6.6-fold decrease), while only less cytotoxicity decreases at 808 nm irradiation (∼28%,∼1.4-fold decrease). Such cytotoxicity studies were consistent with the trends of ^1^O_2_ generation as above, clearly demonstrating the great advantage of deep-tissue penetration of 808 nm (NIR) light for living cell PDT treatment.

### *In vivo* cross-linking of CRUN in tumour-bearing mice

**Figure 3:**
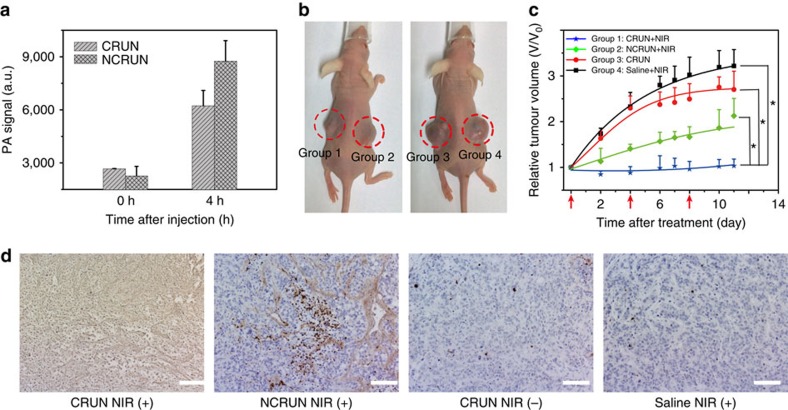
*In vivo* photoacoustic imaging and PDT efficiency by intratumoural injection of CRUN. (**a**) PA signals in tumour region after injection of CRUN (3 mg in 100 μl saline). (**b**) Photographs of tumour-bearing mice after 11 days treatment with CRUN (group 1), NCRUN (group 2), CRUN without NIR light irradiation (group 3) and saline (group 4). (**c**) Relative tumour volumes in the treated groups after PDT therapy under 808 nm laser irradiation. The treatment after 4 h of intratumoural injection (3 mg in 100 μl saline) was started on day 0 and repeated twice at day 4 and day 8 (arrows) followed by 808 nm laser irradiation. (**d**) TUNEL histology of tumour tissues after NIR light-mediated tumour treatment. Scale bar, 100 μm. Date are means±s.d. (*n*=8 mice per group). Statistical significance is assessed by a Student's *t*-test (heteroscedastic, two-sided). *P<0.05.

Inspired by the promising imaging and PDT results in living cells, we examined the *in vivo* theranostic efficacy via intratumoural injection of CRUN into living female Balb/c nude mice. We implanted HT-29 cells (CtsB overexpressing) into the left and right flanks of nude mice. At 2–3 weeks post-implantation, the tumours grew to 3–5 mm in diameter and the living mice underwent photoacoustic imaging and PDT studies. Typically, one group of xenograft mice bearing two tumours were subjected to CRUN and controlled NCRUN conjugates, respectively (3 mg in 100 μl saline), followed by photoacoustic imaging. As compared with the photoacoustic intensity before injection, the mice exhibited a significant increase in photoacoustic signals at 4 h post-injections for both conjugates. Moreover, the photoacoustic signals in the tumour injected with CRUN were found lower (∼30% decrease in intensity) than that subjected to NCRUN (Fig. 3a and Supplementary Fig. 19). These results suggested the feasibility of the enzyme processing to trigger the localization of CRUN at the tumour site and it was the covalent cross-linking that led to the decreased photoacoustic signals.

Furthermore, we also investigated the therapeutic effect of CRUN as a PDT agent for tumour therapy in living mice. As shown in Fig. 3b, in group 1 (*n*=8), CRUN was injected into the implanted tumour on the left flank, and followed by an 808 nm laser irradiation at the tumours after 4 h injection. As contrast, group 2 (*n*=8) mice were intratumourally injected with the control NCRUN on the right flank and subsequent light exposure under identical conditions. Tumour-bearing mice with direct injection of CRUN but no NIR light irradiation were assigned as group 3 (*n*=8). The last group (group 4, *n*=8) consisted of mice injected with saline only. The tumour sizes in the different groups were measured to assess the PDT efficacy over a period of 2 weeks. After CRUN-mediated PDT treatment (*P*=0.038), the tumour growth in group 1 had been significantly inhibited as compared with that observed in group 2 with control NCRUN (*P*=0.033, Fig. 3c), indicating that the enzyme-triggered cross-linking of UCN particles at the tumour region had an important role in enhancing therapeutic outcomes. On the other hand, in group 3 (*P*=0.021) and 4, the tumour sizes grew exponentially over the period of time, suggesting that the treatment with 808 nm laser only or CRUN without light irradiation induced minimum inhibition of tumour growth. To further evaluate the therapeutic efficacy, we also performed TUNEL staining on the tissue sections from different groups after 11 days of PDT treatment (Fig. 3d and Supplementary Fig. 20). As expected, the treated tumours in group 1 mice displayed significant damage than those in group 2 mice. The mice treated with NIR light alone (group 4) and probe without light (group 3) displayed no detectable damage. This is in good accordance with the trends observed in tumour inhibition after NIR light-mediated PDT treatment. More importantly, there were no significant burnt scars on the tumour skins in similar light-mediated treatment by conventionally used 980 nm laser illuminations, clearly implying that the novel enzyme-responsive CRUN conjugates with laser irradiation at 808 nm could serve as a reliable platform for effective *in vivo* antitumour treatment.

### *In vivo* PDT treatment with affinity ligand-modified CRUN

**Figure 4:**
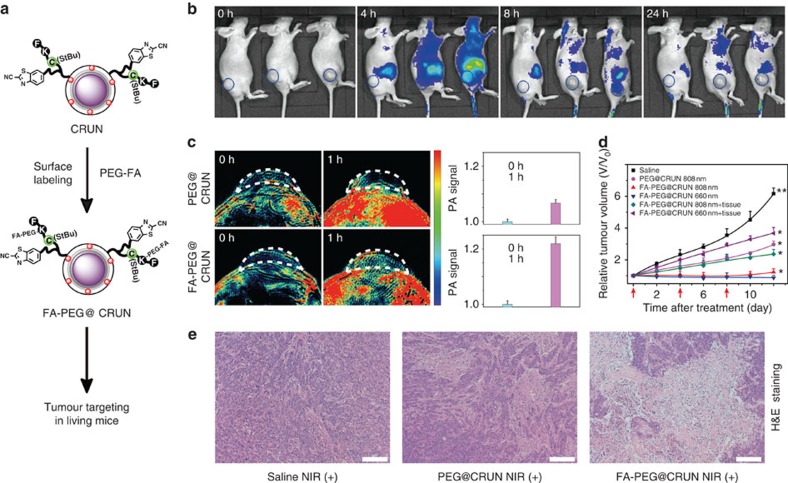
*In vivo* targeted tumour imaging and PDT therapy by intravenous injection of FA-PEG@CRUN. (**a**) Illustration of the targeting strategy of FA-PEG@CRUN through the conjugation of FA and PEG_5000_ on CRUN. (**b**) Fluorescence imaging of tumours (blue circle) in living mice at different time intervals after injection (from left to right: Saline, PEG@CRUN, FA-PEG@CRUN, respectively; λ_ex_=640 nm, λ_em_=670 nm). (**c**) PA imaging signals in the tumour region at different time intervals after intravenous injection (top: PEG@CRUN, bottom: FA-PEG@CRUN, 3 mg in 100 μl saline). (**d**) Tumour volumes change as a function of time in treated groups to evaluate the effectiveness of PDT treatment *in vivo*. The PDT treatment was carried out after 4 h drug-light interval of intravenous injection (3 mg in 100 μl saline) and periodically repeated at indicated time points (arrows) following by 808 nm laser irradiation every two days. (**e**) H&E histology of tumour tissues after 808 nm mediated tumour treatment by intravenous injection. Scale bar, 200 μm. Date are means±s.d. (*n*=8 mice per group). Statistical significance is assessed by a Student's *t*-test (heteroscedastic, two-sided). *P<0.05, **P<0.01.

To further improve the tumour uptake and PDT efficiency, we conjugate an affinity ligand, folic acid (FA), to the surface of CRUN for their recognition to folate receptors that overexpress in most tumours. Meanwhile, considering the fact to enhance the biocompatibility of CRUN in living system, the FA was first coupled with polyethene glycol (PEG_5000_) spacer containing amino and thiol group at each terminus. Such FA-PEG_5000_-SH linkers were then coupled to enzyme-responsive CRUN conjugate through NHS-dPEG_2_-Mal as a linkage moiety to afford final product of FA-PEG@CRUN (Fig. 4a and Supplementary Fig. 21). Upon reaction with CtsB enzyme, FA-PEG@CRUN indicated similar luminescence enhancement as compared with previous CRUN platform without FA modification, suggesting that introduction of FA and PEG linker into CRUN platform would not influence its activity for enzyme recognition (Supplementary Fig. 22).

To study whether FA conjugation can aid tumour uptake in living mice, we used fluorescence imaging to real-time monitor the targeting effect *in vivo*. The female Balb/c nude mice bearing HT-29 tumours were divided into three groups. Group 1 consisted of the mice with intravenous injection of saline. The mice subjected to PEG-modified CRUN particles but no FA ligands were assigned to group 2. In group 3, the tumour-bearing mice were injected with FA-PEG@CRUN via the tail vein. The fluorescent images were recorded at different time intervals after the first injection. Relative to control studies, a significant increase in fluorescence was observed in living mice at 4 h post-injection of probes (Fig. 4b and Supplementary Fig. 23). In contrast, the tumours in group 3 with injection of FA-PEG@CRUN displayed stronger fluorescence than that in group 2 subjected to PEG@CRUN injection. Both fluorescence signals gradually decreased 24 h post-administration through the process of circulation. In addition, similar tumour targeting was also validated by photoacoustic imaging in living mice. After administration of CRUN conjugates at 1 h, the mice showed obvious photoacoustic signal increase in tumours. Moreover, the observed photoacoustic signals in the tumours of group 3 were higher than that in group 2 (Fig. 4c). The higher fluorescence and photoacoustic signals present in tumours confirmed the possible efficacy of the FA ligand in improving the targeting of CRUN conjugates at the tumour region.

Encouraged by the imaging data that revealed the effective tumour uptake of CRUN particles, we continued the light-mediated PDT therapy via intravenous injection of surface-modified CRUN conjugates or saline into six different groups of mice, and *in vivo* tumour inhibition effect was evaluated upon 808 or 660 nm light illumination. As shown in Fig. 4d, after 808 nm laser illumination, the tumour sizes in group 2 (*n*=8, *P*=0.050, with injection of PEG@CRUN) and 3 (*n*=8, *P*=0.038, injected with FA-PEG@CRUN) were found to be reduced significantly over the therapeutic period, whereas the tumour sizes in control group 1 (*n*=8) with injection of saline revealed a fast growth rate. In addition, during the treatment, the tumours in group 3 displayed more effective inhibition than those in group 2. These data suggested that the facilitated tumour uptake by FA ligands and effective tumour reduction was mainly from the production of ^1^O_2_ from Ce6 anchored on the CRUN surface.

Notably, direct 808 nm (group 3) and 660 nm (group 5, *P*=0.008) laser irradiation of living animals with intravenous injection of FA-PEG@CRUN resulted in comparable PDT efficiency, in which tumour growth has been greatly suppressed (Fig. 4d). However, considering the different light penetration, similar *in vivo* PDT treatments were also performed when the tumours were covered with a 2-mm thick pork tissue. Compared with mice with FA-PEG@CRUN injection and laser illumination at 808 nm (group 4, *P*=0.019), the presence of pork tissue could significantly compromise tumour therapy for the mice treated with FA-PEG@CRUN and 660 nm laser excitation (group 6, *P*=0.029), further demonstrating the benefits of deep tissue penetration in 808 nm laser-mediated tumour treatment.

Besides the promising antitumour therapy *in vivo*, the histological tumour tissues analysis also revealed the efficacy of PDT treatment. After 12 days of light-mediated PDT at 808 nm, hematoxylin and eosin and TUNEL staining of tumour tissues displayed more significant damage in the mice with intravenous injection of FA-PEG@CRUN (group 3) than those tumours treatment in control groups (Fig. 4e and Supplementary Fig. 24). Moreover, there was no obvious damage observed in other organs during the treatment (Supplementary Fig. 25). And our inductively coupled plasma spectral analysis also validated more Y^3+^ ion distribution in the tumour region (Supplementary Fig. 26). These results clearly indicated that CRUN conjugates modified with active targeting agents can significantly improve the therapeutic efficacy of *in vivo* tumour treatment with a desirable PDT outcome.

## Discussion

Currently, nanomedicine based on lanthanide-doped UCNs towards cancer imaging and therapy has received considerable attention due to their intrinsic photon-converting nature upon exposure to long-wavelength light irradiation^21^,^22^,^23^,^24^,^25^,^26^,^27^,^28^,^29^. Despite such exceptional optical capabilities in the NIR ranges offered by UCN nanoparticles, the ability to provide specific targeting strategy for effective localization of UCNs within pathological areas, especially at the tumour site, remains a critical challenge. So far, the ligand-mediated recognition strategies based on the conjugation of affinity moieties to the particle surface have been mostly applied to achieve tumour targeting specificity^30^,^31^,^32^,^33^,^34^. Considering the complex and dynamic physiological cell conditions, the results along with the stochastic nature of ligand–receptor interactions between normal and tumour cells are not so satisfactory. Alternative to develop ‘smart' conjugating nanoplatforms that are sensitive to local tumour environment conditions (for example, a particular pH, temperature or enzyme level and so on), and then facilitate specific localization of UCN particles at tumour sites would be thus a highly desirable option^10^,^11^.

In this study, by exploiting the advantages of intrinsic enzymatic stimulus to specifically target the tumour areas, we developed such a ‘smart' upconversion nanoconjugate that can selectively react with protease enzyme CtsB, which has been considered as one important biomarker in many types and stages of cancers^37^,^44^. Upon tumour-specific enzymatic CtsB reaction, the CtsB-sensitive peptide on the UCN particle surface can be cleaved, which exposes free cysteine and induces its condensation reaction with CBT groups on the surface of neighbouring particles, therefore triggering the covalent cross-linking of UCNs particles. Our extensive *in vitro* and *in vivo* fluorescence and photoacoustic imaging studies have validated the successful enzyme-triggered cross-linking reaction, which easily recognized the enhanced light converting emission and unique targeting of enzyme-sensitive UCNs at tumour sites.

So far, photodynamic antitumour therapy has been regarded as a minimally invasive treatment modality, and has been widely applied in clinics for various types of cancer treatment^28^,^30^,^32^. Compared with conventional chemotherapy, light-mediated PDT exhibits some unique advantages, in which cytotoxic photosensitizers can be selectively activated by manipulating the location of light exposure. Currently, the most clinically used photosensitizers are excited at a shorter wavelength range from 630 to 700 nm, which unfortunately has limited penetration depth in biological tissues. One promising strategy to avoid this limitation is to shift the excitation wavelength to the NIR region (700–1,000 nm), in which biological tissues have maximum light transparency, and thus ideal for *in vivo* optical imaging and phototherapy^35^,^36^. Actually, latest advances in rare-earth doped UCNs have witnessed unique upconverting optical properties, which can transduce low-energy NIR light illumination into high-energy emissions from ultraviolet to visible range. Such exceptional light-converting capabilities could greatly match the demands and thus achieve deeper tissue penetration to benefit biomedical applications including photodynamic therapy, biological imaging and remotely controlled release of therapeutic agents *in vitro* and *in vivo*^5^,^27^,^28^,^29^,^30^,^32^.

Current progress of UCN-based PDT system mostly utilizes the NIR light excitation wavelength at 980 nm, at which, however, the absorption of water is significant^27^,^28^,^29^. Such 980 nm light-mediated PDT is facing tremendous hurdles to travel into deeper tissue and bypass the inevitable heat effect^30^,^31^,^32^,^33^,^34^. Therefore, efforts have been intensively made to adjust the excitation range of UCNs to a shorter wavelength to meet the demands of the medical spectral window (that is, ∼700–900 nm) (refs 35, 36). Very recently, Nd^3+^-doped upconversion processes based on a new excitation wavelength around 808 nm have been investigated to manipulate optical properties of lanthanide UCNs to minimize this photothermal effect^26^,^36^. By taking this promising advantage, in this study, we have carried out a series of UCN-based PDT experiments, and systematically compared the differences of photodynamic antitumour effect *in vitro* and *in vivo* through 808 and 660 nm laser irradiation. Our data clearly demonstrated that the PDT treatment efficacy upon 808 nm light illumination did not show significant decrease when the pork tissue (for example, 2 mm thickness) was used to increase the tissue depth; however, the dramatic drop of therapeutic effect was observed in the similar treatment under 660 nm laser excitation (Figs 2i and 4d, and Supplementary Fig. 18), suggesting that 808 nm light-mediated UCNs exhibit deeper tissue penetration and better antitumour effect than those in the conventional PDT treatment system. Meanwhile, during the process of effective UCN-based PDT treatment, the prolonged 808 nm laser irradiation would not induce significant overheating and tissue damage, thus providing promising prospect for their safer applications in the future.

More significantly, in response to specific enzyme stimulation within tumours, our unique UCNs platform could undergo covalent cross-linking reactions and enable the selective accumulation of particles at the tumour region. The *in situ* enzyme-triggered interparticle cross-linking exhibited enhanced light converting emission and could amplify the singlet oxygen generation. Both our *in vitro* and *in vivo* theranostic studies successfully demonstrated that such unique enzyme-responsive localization of light-converted rare-earth nanostructures could significantly inhibit the tumour growth in process of PDT treatment as compared with those UCNs that could not undergo cross-linking reaction.

In summary, a novel targeting strategy based on specific enzymatic reaction was presented to effectively localize light converting UCN nanocarriers at tumour area. Such tumour microenvironment-responsive enzyme reactions would trigger the covalent cross-linking of single UCN particles, and thus display unique particle localization to facilitate promising multimodality imaging and amplified PDT therapy of malignant tumours *in vitro* and *in vivo* upon NIR light illumination at 808 nm. This well-defined rational design could be easily extended into other unique functional materials and open new doors for precision nanomedicine in the future clinical applications.

## Methods

### General

Synthetic procedures and chemical characterizations of all the probes are described in the Supplementary Methods.

### *In vitro* fluorescence imaging in living cells

The human colorectal adenocarcinoma cell line (HT-29, cat. no. HTB-38) and mouse embryonic fibroblast cell line (NIH/3T3, cat. no. CRL-1658) were provided from American type culture collection and checked for mycoplasma contamination. These cells are not listed by ICLAC as misidentified cell lines (3 October 2014). CtsB-overexpressing HT-29 cells and CtsB-deficient NIH/3T3 cells were seeded with a cell density of 1 × 10^5^ in an ibidi confocal μ-dish (35 mm, plastic bottom) in 1 ml DMEM media which was incubated for 24 h at 37 °C. The seeded cells were subsequently treated with CRUN (50 μg ml^−1^) and incubated for 4 h. The cell nuclei were stained by DAPI (1 μg ml^−1^) for 20 min and then the cells were washed with PBS (pH 7.4) for three times. Control experiments were setup by treating the two cell lines with CRUN in the presence of CtsB inhibitor and controlled NCRUN conjugates. For the cell-inhibition assay, the cells were first pretreated with CtsB inhibitor (antipain hydrochloride, 100 μM in DMEM) for 2 h at 37 °C followed by the addition of CRUN and incubated for further 4 h. Fluorescence imaging of the samples were then carried out under confocal microscopy system using a continuous-wave 980 nm laser as excitation source (EINST Technology). The quantification of CtsB enzyme expression in HT-29 and NIH/3T3 cells were performed by Human CtsB ELISA kit according to the standard procedure described by the manufacturer (CUSABIO, USA).

### *In vivo* animal studies

All animal experimental procedures were performed in accordance with the protocol #120774 approved by the Institutional Animal Care and Use Committee (IACUC). Female Balb/c nude mice (∼6–8-weeks old) were purchased from Charles River Laboratories (Shanghai, China). Xenograft mice models were established by injecting 0.1 ml of tumour cells suspension (PSB/matrigel, BD biosciences, 1:1 v/v) containing 3 × 10^6^ HT-29 cells into both flanks or only right side of mouse. When the tumour volume reached a palpable size, the mouse was used for further studies.

### *In vivo* cross-linking of CRUN in tumour-bearing mice

Xenograft mice models were treated when the tumour volumes approached 3–5 mm in diameter. For *in vivo* PDT treatment, the tumour-bearing mice were randomized into four groups (*n*=8, each group) and treated by intratumoural injection of particle samples. In group 1, CRUN (3 mg in 100 μl of saline) was directly injected into implanted tumours, followed by an 808 nm laser irradiation after 4 h injection. In group 2, same amounts of controlled NCRUN conjugates were injected with subsequent laser exposure. Control experiments with injection of CRUN but no NIR light irradiation (group 3) and saline with laser treatment (group 4) were used, respectively. The effective exposure time for each mouse was 45 min (5 min interval after 5 min irradiation with total treatment time of 90 min) with a power density at 0.4 W cm^−2^ (fluence 1,080 J cm^−2^). A second- and third-dose injection followed by PDT treatment described above was repeated at 4 days and 8 days after the first-dose injection, respectively. Tumour size was measured three times a week using a vernier caliper upon PDT treatment. The tumour volume was calculated using the following equation: tumour volume (V)=length × width^2^/2. Relative tumour volume was calculated as V/V_0_ (V_0_ was the initial tumour volume before PDT treatment).

### *In vivo* targeted PDT treatment with intravenous injection

Xenograft HT-29 tumour model on the right side of female Balb/c mice were first developed as described above. For *in vivo* tumour targeting PDT treatment, the tumour-bearing mice had been randomly arranged into six different groups (*n*=8, each group) to perform a series of intravenous injections of saline (group 1), PEG@CRUN (group 2) and FA-PEG@CRUN (group 3–6), respectively (3 mg in 100 μl saline). After 4 h of intravenous injection, laser treatment was performed on groups 1–6 by irradiating the tumour region with a continuous 808 or 660 nm laser. The effective exposure time for each mouse was 45 min (5 min interval after 5 min irradiation with a total treatment time of 90 min) with a power density at 0.4 W cm^−2^ (fluence 1,080 J cm^−2^). The tumours in group 4 and 6 were covered by 2-mm thick pork tissue and then irradiated with 808 or 660 nm lasers under the same condition. The PDT treatment with laser irradiation was repeated every 2 days. A second- and third-dose injection of the PDT treatment described above was repeated at 4 days and 8 days after the first-dose injection, respectively. Tumour size was measured and calculated as described above.

## Additional information

**How to cite this article:** Ai, X. *et al*. *In vivo* covalent cross-linking of photon-converted rare-earth nanostructures for tumour localization and theranostics. *Nat. Commun.* 7:10432 doi: 10.1038/ncomms10432 (2016).

## Supplementary Material

Supplementary Information Supplementary Figures 1-26, Supplementary Methods and Supplementary References

## References

[b1] Gao Y., Shi J., Yuan D. & Xu B. Imaging enzyme-triggered self-assembly of small molecules inside live cells. Nat. Commun. 3, 1033–1040 (2012). 22929790 10.1038/ncomms2040 PMC3521559

[b2] Williams R. J. . Enzyme assisted self-assembly under thermodynamic control. Nat. Nanotechnol. 4, 19–24 (2009). 19119277 10.1038/nnano.2008.378

[b3] Lovell J. F. . Porphysomenanoesicles generated by porphyrin bilayers for use as multimodal biophotonic contrast agents. Nat. Mater. 10, 324–332 (2011). 21423187 10.1038/nmat2986

[b4] Petros R. A. & Desimone M. J. Strategies in the design of nanoparticles for therapeutic applications. Nat. Rev. Drug Discov. 9, 615–627 (2010). 20616808 10.1038/nrd2591

[b5] Mitragotri S., Burke P. A. & Langer R. Overcoming the challenges in administrating biopharmaceuticals: formulation and delivery strategies. Nat. Rev. Drug Discov. 13, 655–672 (2014). 25103255 10.1038/nrd4363 PMC4455970

[b6] Cheng C. J., Tietjen G. T., Saucier-sawyer J. K. & Saltzman W. M. A holistic approach to targeting disease with polymeric nanoparticles. Nat. Rev. Drug Discov. 14, 239–247 (2015). 25598505 10.1038/nrd4503 PMC4451203

[b7] Davis M. E., Chen Z. G. & Shin D. M. Nanoparticle therapeutics: an emerging treatment modality for cancer. Nat. Rev. Drug Discov. 7, 771–882 (2008). 18758474 10.1038/nrd2614

[b8] Giljohann D. A. & Mirkin C. A. Drivers of biodiagnostics development. Nature 580, 461–464 (2009). 19940916 10.1038/nature08605 PMC3936986

[b9] Koo H. . *In vivo* targeted delivery of nanoparticles for theranosis. Acc. Chem. Res. 44, 1018–1028 (2011). 21851104 10.1021/ar2000138

[b10] Yu M. K., Park J. & Jon S. Y. Targeting strategies for multifunctional nanoparticles in cancer imaging and therapy. Theranostics 2, 3–44 (2012). 22272217 10.7150/thno.3463 PMC3263514

[b11] Thaker A. S. & Gambhir S. S. Nanooncology: the future of cancer diagnosis and therapy. Cancer J. Clin. 63, 395–418 (2013). 10.3322/caac.21199 24114523

[b12] Mura S., Nicolas J. & Couvreur P. Stimuli-responsive nanocarriers for drug delivery. Nat. Mater. 12, 991–1003 (2013). 24150417 10.1038/nmat3776

[b13] Liang G., Ren H. & Rao J. A biocompatible condensation reaction for controlled assembly of nanostructures in living cells. Nat. Chem. 2, 54–60 (2010). 21124381 10.1038/nchem.480 PMC3196337

[b14] Haun J. B., Devaraj N. K., Hilderbrand S. A., Lee H. & Weissleder R. Bioorthogonal Chemistry amplifies nanoparticles binding and enhances the sensitivity of cell detection. Nat. Nanotechnol. 5, 660–665 (2010). 20676091 10.1038/nnano.2010.148 PMC2934903

[b15] Tsukiji S., Miyagawa M., Takaoka Y., Tamura T. & Hamachi I. Ligand-directed tosyl chemistry for protein labeling *in vivo*. Nat. Chem. Biol. 5, 341–343 (2009). 19330012 10.1038/nchembio.157

[b16] Laughlin S. T., Baskin J. M., Amacher S. L. & Bertozzi C. R. *In vivo* imaging of membrane-associated glycans in developing zebrafish. Science 320, 664–667 (2008). 18451302 10.1126/science.1155106 PMC2701225

[b17] Prescher J. A., Dube D. H. & Bertozzi C. R. Chemical remodelling of cell surfaces in living animals. Nature 430, 873–877 (2004). 15318217 10.1038/nature02791

[b18] Ye D. . Bioorthongonal cyclization-mediated *in situ* self-assembly of small-molecule probes for imaging caspase activity *in vivo*. Nat. Chem. 6, 519–526 (2014). 24848238 10.1038/nchem.1920 PMC4031611

[b19] Lang K. & Chin J. W. Cellular incorporation of unnatural amino acids and bioorthogonal labeling of proteins. Chem. Rev. 114, 4764–4806 (2014). 24655057 10.1021/cr400355w

[b20] Grammel M. & Hang H. C. Chemical reporters for biological discovery. Nat. Chem. Biol. 9, 475–484 (2013). 23868317 10.1038/nchembio.1296 PMC3866016

[b21] Deng R. . Temporal full-colour tuning through non-steady-state upconversion. Nat. Nanotechnol. 10, 237–242 (2015). 25599189 10.1038/nnano.2014.317

[b22] Feng W., Zhu X. & Li F. Recent advances in the optimization and functionalization of upconversion nanomaterials for *in vivo* bioapplications. NPG Asia Mater. 5, e75 (2013).

[b23] Yang Y. . *In vitro* and *in vivo* uncaging and bioluminescent imaging through photocaged upconversion nanoparticles. Angew. Chem. Intl. Ed. 51, 3125–3127 (2012). 10.1002/anie.201107919 22241651

[b24] Idris N. M. . *In vivo* photodynamic therapy using upconversion nanoparticles as remote-controlled nanotransducers. Nat. Med. 18, 1580–1585 (2012). 22983397 10.1038/nm.2933

[b25] Yang D. . Current advances in lanthanide ion (Ln^3+^)-based upconversion nanomaterials for drug delivery. Chem. Soc. Rev. 44, 1416–1448 (2015). 24988288 10.1039/c4cs00155a

[b26] Xie X. J. . Mechanistic investigation of photon upconversion in Nd^3+^-sensitized core-shell nanoparticles. J. Am. Chem. Soc. 135, 12608–12611 (2013). 23947580 10.1021/ja4075002

[b27] Kachynski A. V. . Photodynamic therapy by *in situ* nonlinear photon conversion. Nat. Photon. 8, 455–461 (2014).

[b28] Xu C. T. . Upconverting nanoparticles for pre-clinical diffuse optical imaging, microscopy and sensing: current trends and future challenges. Laser Photon. Rev. 7, 663–697 (2013).

[b29] Zou W., Visser C., Maduro J. A., Pshenichnikov M. S. & Hummelen J. C. Broadband dye-sensitized upconversion of near-infrared light. Nat. Photon. 6, 560–564 (2012).

[b30] Wang C., Cheng L. & Liu Z. Drug delivery with upconversion nanoparticles for multi-functional targeted cancer cell imaging and therapy. Biomaterials 32, 1110–1120 (2011). 20965564 10.1016/j.biomaterials.2010.09.069

[b31] Zhou L. . Single-band upconversion nanoprobes for multiplexed simultaneous *in situ* molecular mapping of cancer biomarkers. Nat. Commun. 6, 6938 (2015). 25907226 10.1038/ncomms7938 PMC4423208

[b32] Lu S. . Multifunctional nano-bioprobes based on rattle-structured upconverting luminescent nanoparticles. Angew. Chem. Int. Ed. 54, 7915–7919 (2015). 10.1002/anie.201501468 26013002

[b33] Xiao Q. . A core/satellite multifunctional nanotheranostic for *in vivo* imaging and tumor eradication by radiation/photothermal synergistic therapy. J. Am. Chem. Soc. 135, 13041–13048 (2013). 23924214 10.1021/ja404985w

[b34] Li L. . Biomimetic surface engineering of lanthanide-doped upconversion nanoparticles as versatile bioprobes. Angew. Chem. Int. Ed. 51, 6121–6125 (2012). 10.1002/anie.201109156 PMC3516999 22566291

[b35] Ai F. . A core-shell-shell nanoplatform upconverting near-infrared light at 808 nm for luminescence imaging and photodynamic therapy of cancer. Sci. Rep. 5, 10785 (2015). 26035527 10.1038/srep10785 PMC4451683

[b36] Shen J. . Engineering the upconversion nanoparticle excitation wavelength: cascade sensitization of tri-doped upconversion colloidal nanoparticles at 800 nm. Adv. Opt. Mater. 1, 644–650 (2013).

[b37] Lecaille F., Kaleta J. & Bromme D. Human and parasitic papain-like cysteine proteases: their role in physiology and pathology and recent developments in inhibitor design. Chem. Rev. 102, 4459–4488 (2002). 12475197 10.1021/cr0101656

[b38] Wang L. V. & Hu S. Photoacoustic tomography: *in vivo* imaging from organelles to organs. Science 335, 1458–1462 (2012). 22442475 10.1126/science.1216210 PMC3322413

[b39] Nie L. & Chen X. Structural and functional photoacoustic molecular tomography aided by emerging contrast agents. Chem. Soc. Rev. 43, 7132–7170 (2014). 24967718 10.1039/c4cs00086b PMC4569000

[b40] Sheng Y., Liao L. D., Thakor N. & Tan M. C. Rare-earth doped particles as dual-modality contrast agent for minimally-invasive luminescence and dual-wavelength photoacoustic imaging. Sci. Rep. 4, 6562 (2014). 25297843 10.1038/srep06562 PMC4190509

[b41] Chris H. J. H. . Multifunctional photosensitizer-based contrast agents for photoacoustic imaging. Sci. Rep. 4, 5342 (2014). 24938638 10.1038/srep05342 PMC4061552

[b42] Atwater H. A. & Polman A. Plasmonics for improved photovoltaic devices. Nat. Mater. 9, 205–213 (2010). 20168344 10.1038/nmat2629

[b43] Zeng Z., Mizukami S., Fujitab K. & Kikuchi K. An enzyme-responsive metal-enhanced nearinfrared fluorescence sensor based on functionalized gold nanoparticles. Chem. Sci. 6, 4934–4939 (2015). 10.1039/c5sc01850a PMC5664366 29142724

[b44] Talieria M. . Cathepsin B and cathepsin D expression in the progression of colorectal adenoma to carcinoma. Cancer Lett. 205, 97–106 (2004). 15036666 10.1016/j.canlet.2003.09.033

[b45] Lovell J. F., Liu T. W. B., Chen J. & Zheng G. Activatable photosensitizers for imaging and therapy. Chem. Rev. 110, 2839–2857 (2010). 20104890 10.1021/cr900236h

