# Supplementary material for: In vivo covalent cross-linking of photon-converted rare-earth nanostructures for tumour localization and theranostics
Source: Nat Commun. 2016 Jan 20;7:10432. doi: 10.1038/ncomms10432 (PMC4736106; doi:10.1038/ncomms10432)
Supplement: Supplementary Information — Supplementary Figures 1-26, Supplementary Methods and Supplementary References [file ncomms10432-s1.pdf]

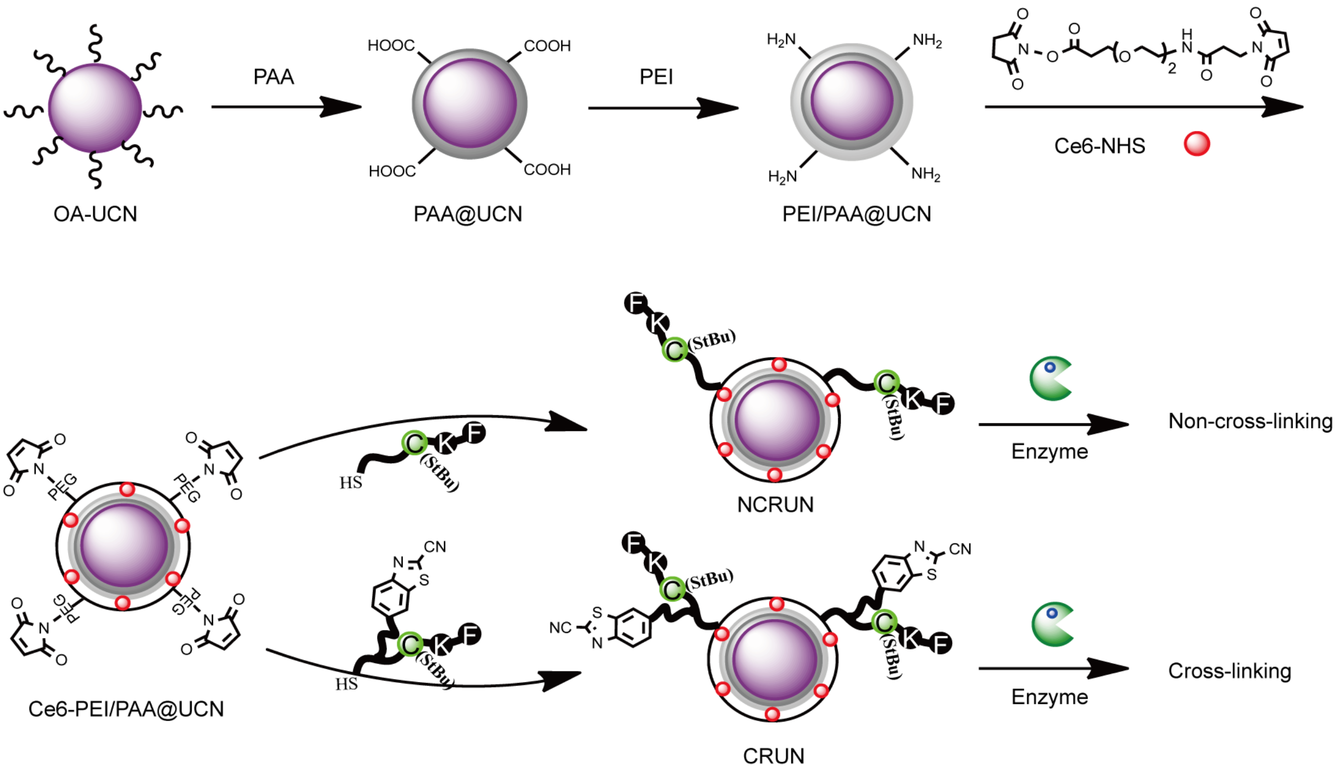

Supplementary Fig. 1. Design and synthesis of enzyme triggered cross-linking of CRUN and control NCRUN.

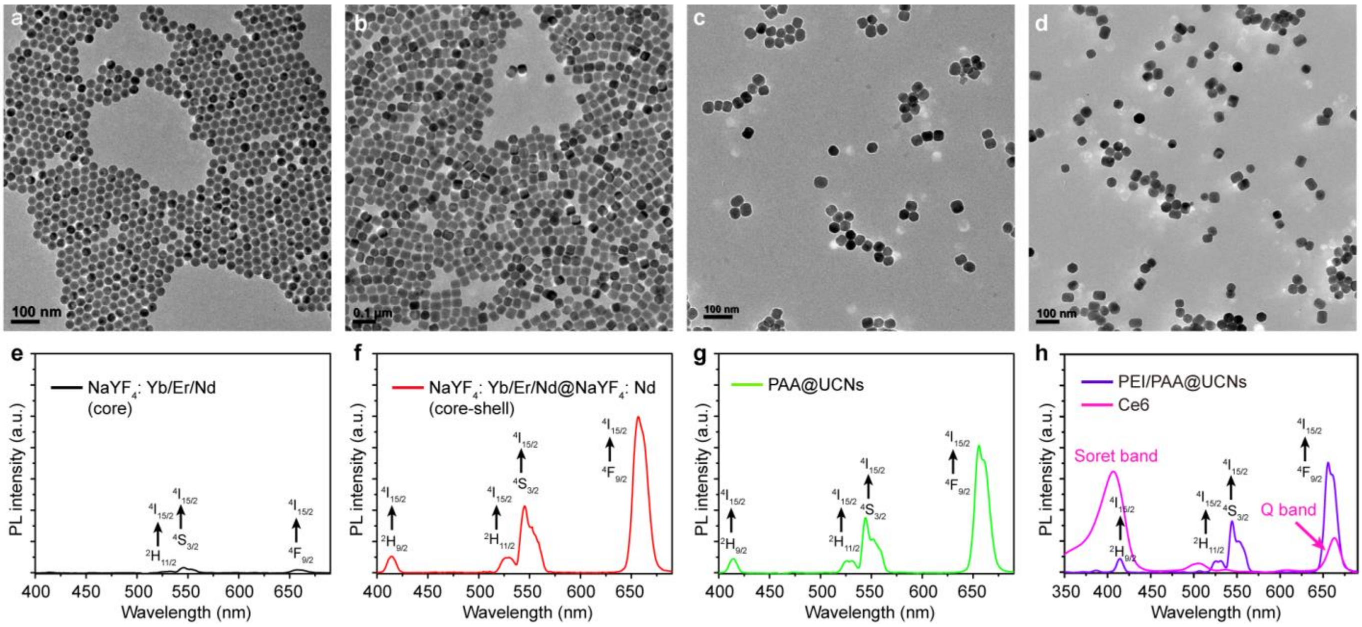

Supplementary Fig. 2. TEM and spectroscopic characterization of rare-earth doped UCNs. TEM images of the as-prepared materials of (**a**) $NaYF_4$:Yb/Er/Nd (18/0.5/1%) core UCNs, (**b**) $NaYF_4$:Yb/Er/Nd (18/0.5/1%) @$NaYF_4$:Nd (20%) core-shell UCNs, (**c**) PAA@UCNs and (**d**) PEI/PAA@UCNs, respectively. Scale bar: 100 nm. Figures (**e**)-(**g**) show the corresponding UCL spectra of core UCNs, core-shell UCNs, PAA@UCNs, respectively. Figure (**h**) shows the corresponding emission spectra of PEI/PAA@UCNs and the UV-Vis absorption of Ce6. The particle concentration of samples of (**e**)-(**h**) is 2 mg mL^{-1}.

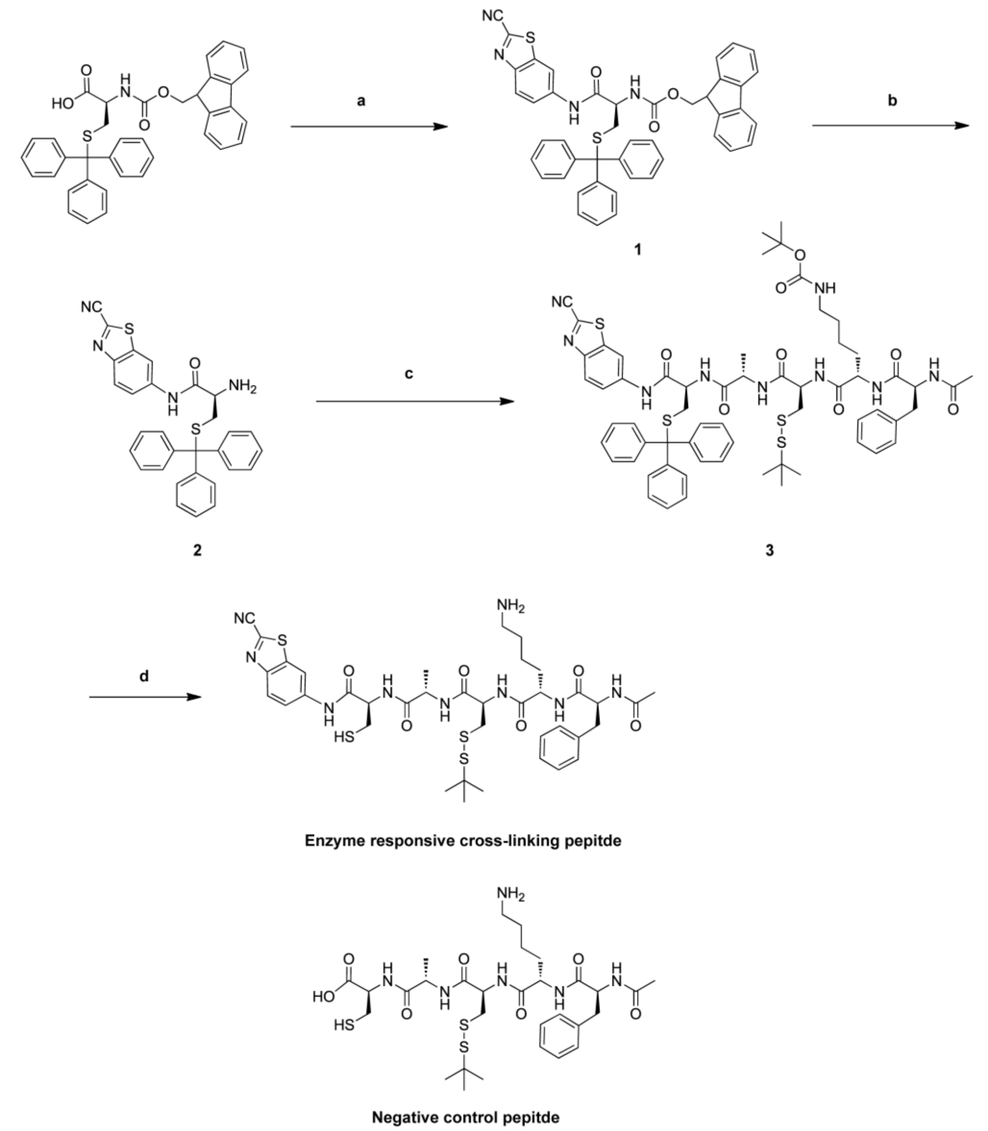

Supplementary Fig. 3. Synthesis of enzyme responsive cross-linking peptide and control non-cross-linking peptide. (**a**) CBT, NMP, IBCF, 0°C-r.t. (**b**) 20% piperidine in DMF. (**c**) Ac-FK(Boc)C(StBu)A-OH, HBTU, DIPEA. (**d**) TFA/TIPS/DCM (95/2.5/2.5%).

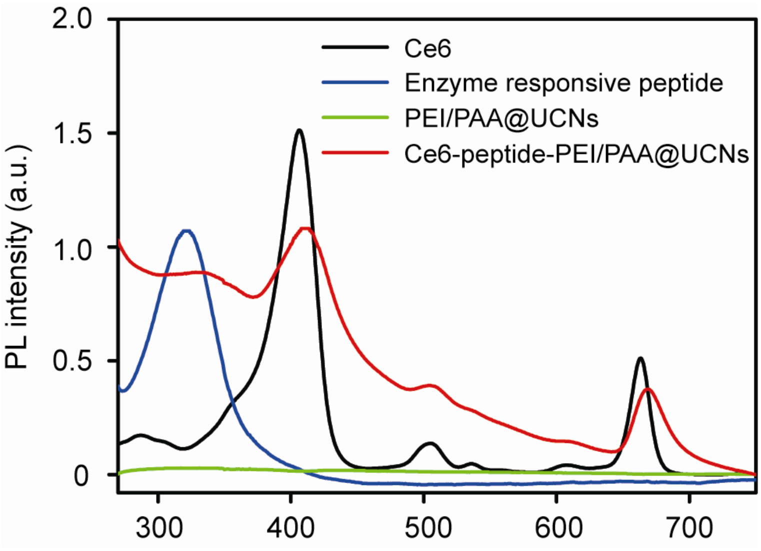

Supplementary Fig. 4. Spectroscopic characterization for the formation of UCNs conjugates with chlorine e6 (Ce6) and enzyme responsive cross-linking peptide. UV-Vis absorption spectra of Ce6, enzyme responsive cross-linking peptide, PEI/PAA@UCNs and Ce6-peptide-PEI/PAA@UCNs (CRUN, 1 mg mL^{-1}) in aqueous solution.

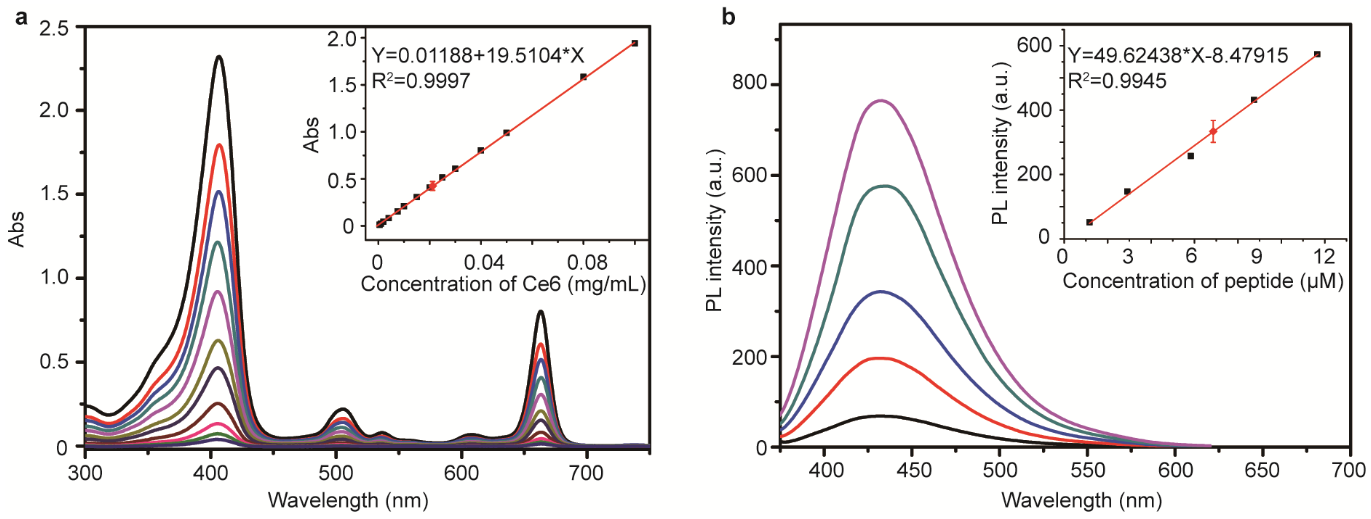

Supplementary Fig. 5. Quantification of chlorine e6 (Ce6) and enzyme responsive cross-linking peptide on UCNs surface. (**a**) UV-Vis absorbance spectra of Ce6 at different concentration (0.84-167 μM). Inset: the standard curve for Ce6 detection at absorbance wavelength of 663 nm. (**b)** Fluorescence spectra of enzyme responsive cross-linking peptide (Ac-FKC(StBu)AC(SH)-CBT) at different concentrations (1 ~ 12 μM). Inset: the standard curve for monitoring of the peptide at the emission wavelength of 450 nm (λ_{ex} = 320 nm excitation). Data bars show mean±s.d. (*n* = 3 technical replicates).

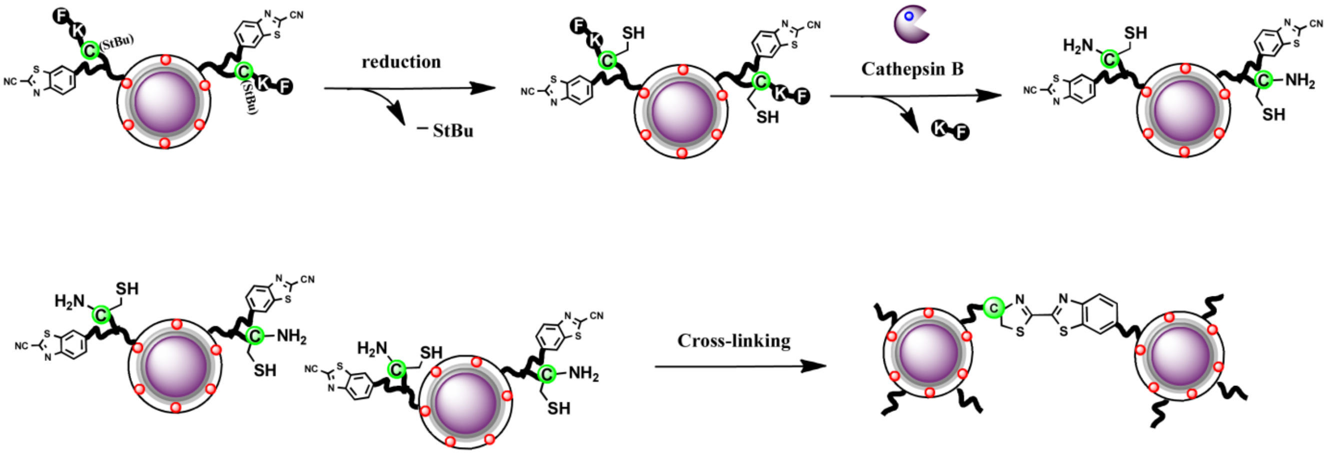

Supplementary Fig. 6. The reaction scheme of enzyme triggered cross-linking of CRUN.

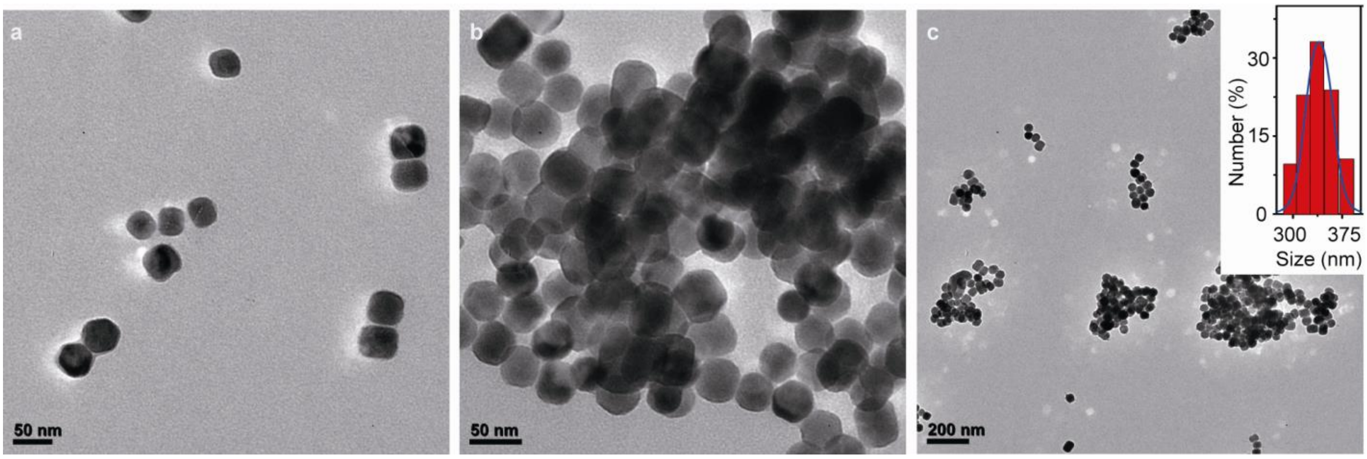

Supplementary Fig. 7. TEM images and DLS characterization of CtsB enzyme triggered cross-linking of CRUN. TEM images show the CRUN (**a**) in the absence of CtsB, (**b**) in the presence of CtsB (55 nM), (**c**) in the presence of CtsB (55 nM) and antipain inhibitor (100 µM) respectively**.** DLS shows the hydrodynamic diameter at about 330 nm.

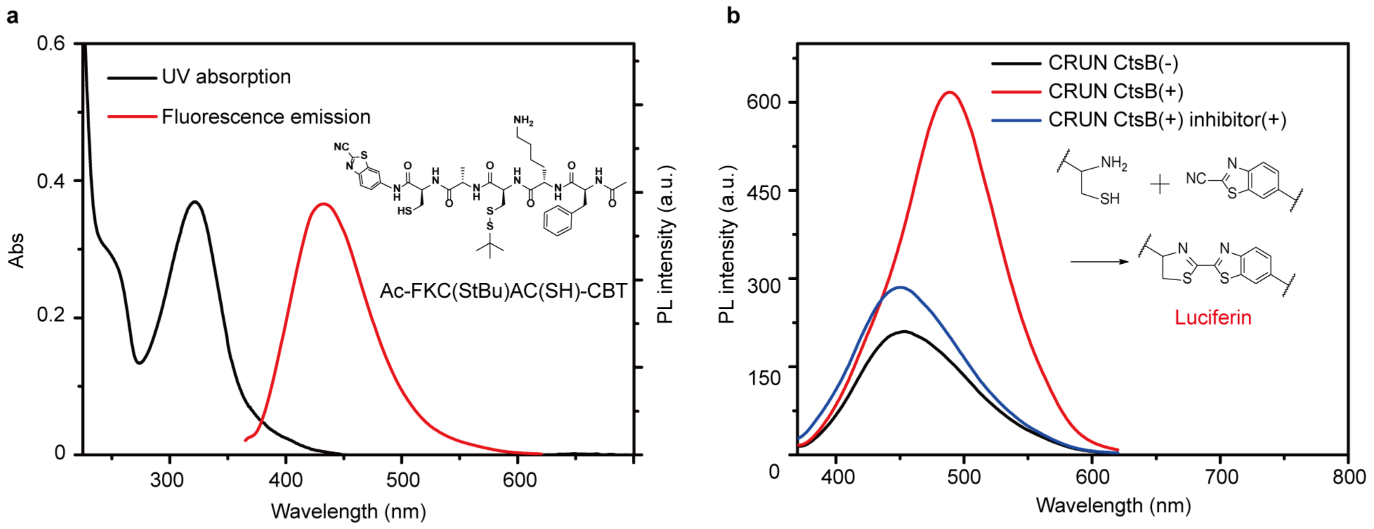

Supplementary Fig. 8. Spectroscopic analysis of CtsB enzyme triggered cross-linking of CRUN. (**a**) UV-Vis absorption and fluorescence spectra of enzyme responsive cross-linking peptide (100 µM) with an absorption peak at 320 nm and a fluorescence emission peak at 450 nm. (**b**) Fluorescence emission of the CtsB (55 nM) treated CRUN (1.5 mg mL^{-1}) red-shifts from 450 nm to 489 nm and fluorescent emission of CRUN (1.5 mg mL^{-1}) in the presence of CtsB and antipain inhibitor (100 µM) shows a slight increase of emission at 450 nm.

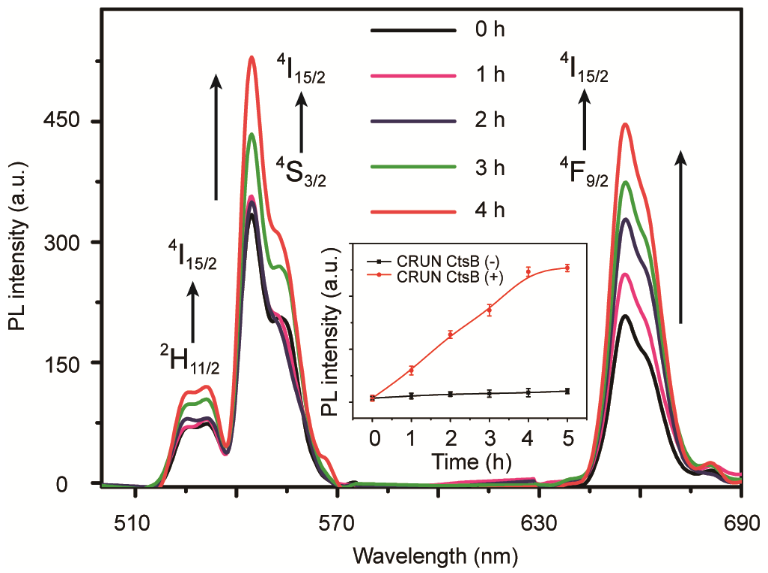

Supplementary Fig. 9. The upconverted luminescence (UCL) of CRUN at different time intervals with CtsB enzyme treatment. Inset: UCL signals at 655 nm during the cross-linking reaction in different time. Data bars show mean±s.d. (*n* = 3 technical replicates).

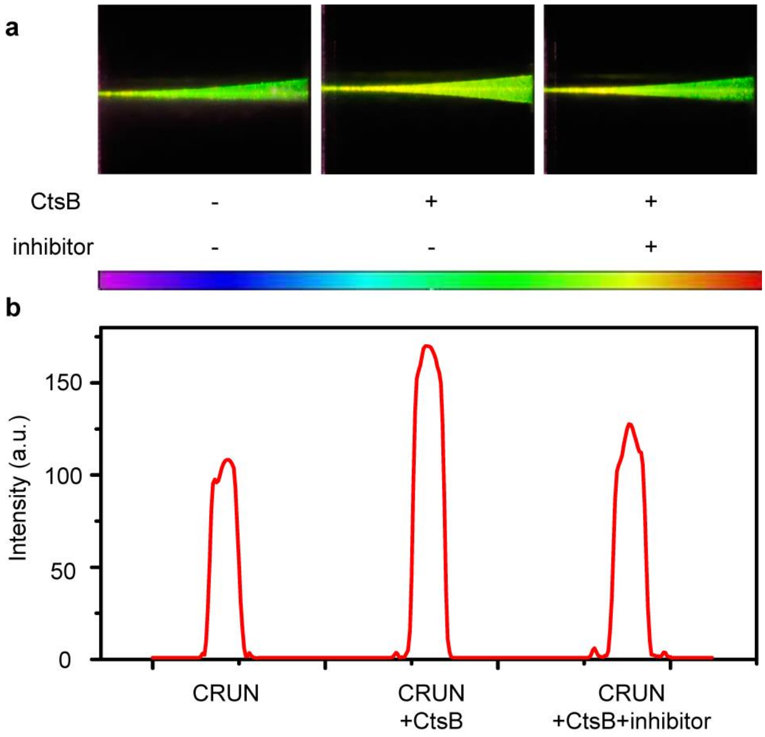

Supplementary Fig. 10. Photoluminescence characterization of cross-linking reaction triggered by tumor specific CtsB enzyme. (**a**) Luminescence photographs of CRUN (1.5 mg mL^{-1}) in the absence of CtsB (left), in the presence of CtsB (middle), and with CtsB and inhibitor treatment (right). (**b**) Fluorescence intensity was measured by Image J. The concentration of CtsB enzyme and antipain inhibitor were 55 nM and 100 µM.

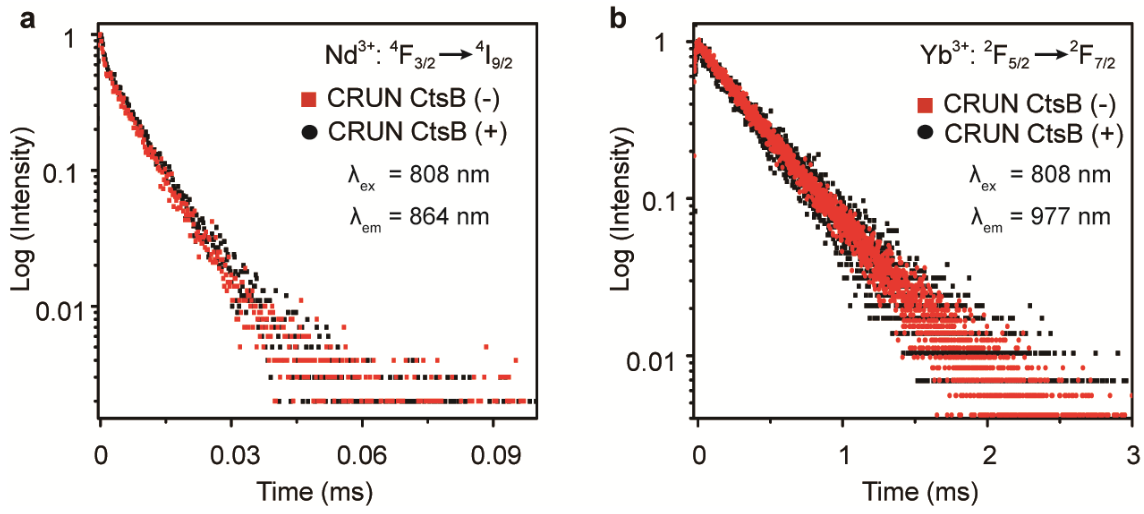

Supplementary Fig. 11. Luminescence decay curves of lanthanide dopants of **(a)** Nd^{3+} (λ_{em} = 864 nm) and **(b)** Yb^{3+} (λ_{em} = 977 nm) in CRUN before and after enzyme triggered cross-linking reaction under 808 nm light irradiation.

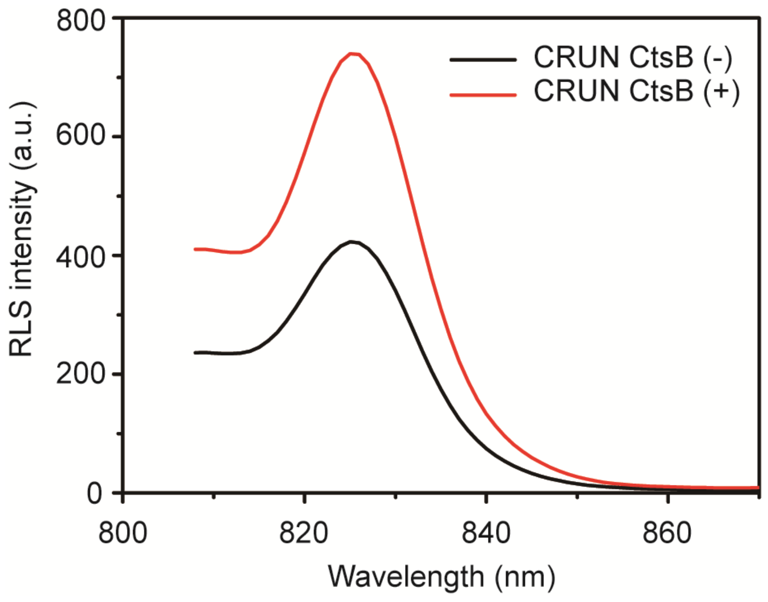

Supplementary Fig. 12. Resonance light scattering (RLS) spectra of CRUN upon tumor specific enzyme treatment.

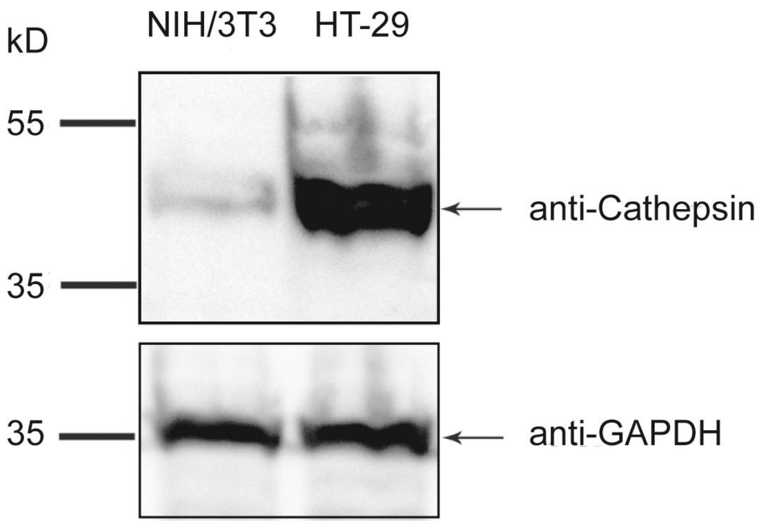

Supplementary Fig. 13. Analysis of cathepsin B enzyme expression by Western blot in HT-29 and NIH/3T3 cells. Anti-GAPDH was included as an internal control.

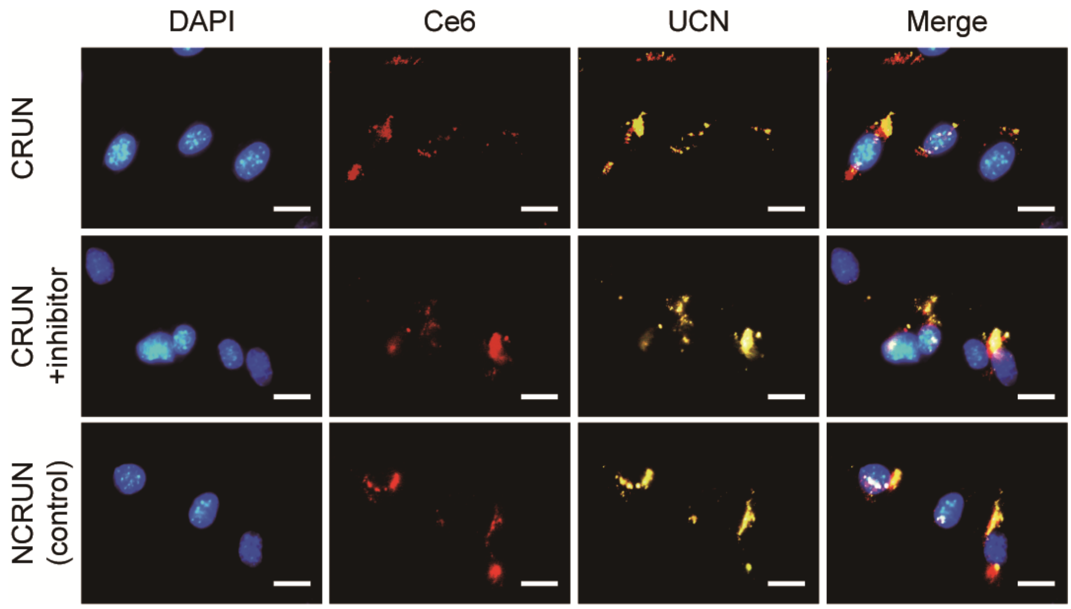

Supplementary Fig. 14. Cellular imaging of enzyme triggered CRUN cross-linking in NIH/3T3 cells. The CRUN (top), CRUN and inhibitor (middle), and the negative control NCRUN (bottom) were incubated with NIH/3T3 cells (50 µg mL^{-1}) at 37 ˚C for 4h. The concentration of inhibitor was 100 µM. Blue: DAPI (λ_{ex}=350 nm/50 nm, λ_{em}=460 nm/50 nm), Red: Ce6 (λ_{ex}=545 nm/25 nm, λ_{em}=610 nm/75 nm), Yellow: UCN (λ_{ex}=980 nm, λ_{em}=350 nm-690 nm). Scale bar: 20 µm.

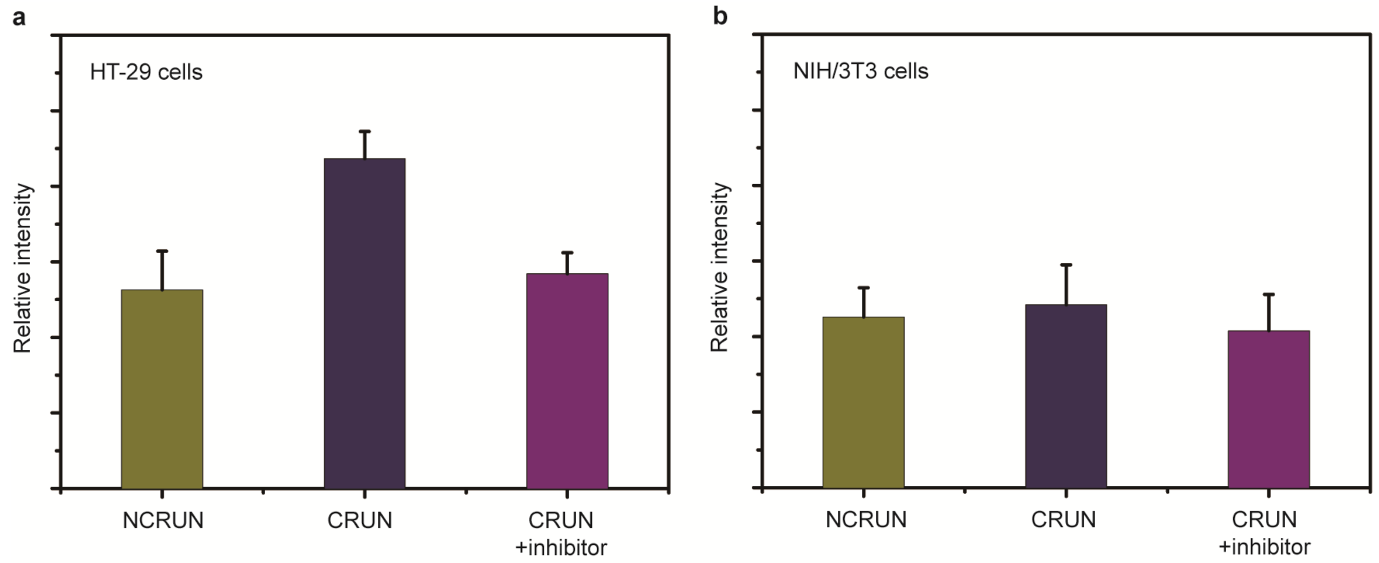

Supplementary Fig. 15. Fluorescence analysis of enzyme triggered particle cross-linking in (a) HT-29 and (b) NIH/3T3 cells. The CRUN and negative control NCRUN (50 μg mL^{-1}) were incubated with two cells at 37 °C for 4 h. The concentration of inhibitor was 100 μM. Fluorescence intensity was measured by Image J. Data bars show mean±s.d. (n = 3 technical replicates).

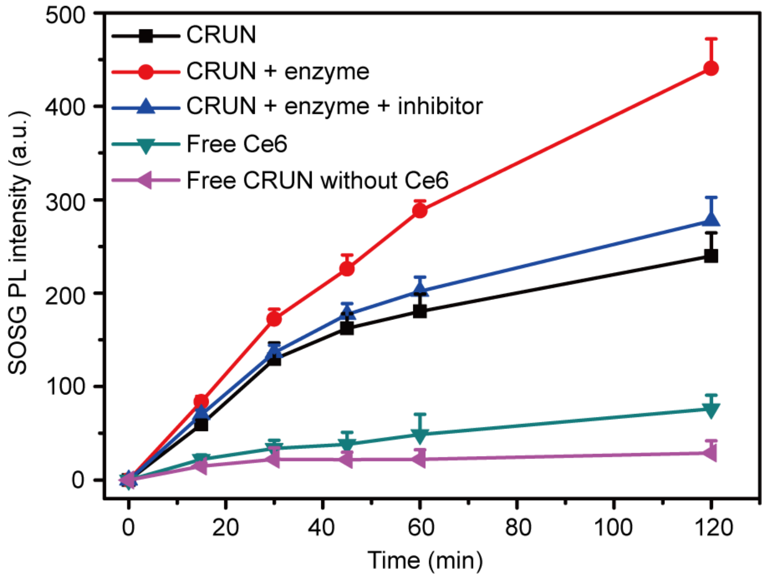

Supplementary Fig. 16. Quantification of singlet oxygen generation from enzyme triggered cross-linking of CRUN upon 808nm laser irradiation. The detection of singlet oxygen generation of CRUN was performed by the singlet oxygen sensor green (SOSG, 2.5 µM) dye following a standard protocol in the presence of laser irradiation (808 nm, 0.4 W cm^{-2}). The concentration of Ce6 in all the samples was 1 µM in PBS (10 mM). The fluorescence intensity of these samples (λ_{ex}= 494 nm, λ_{em}= 530 nm) was recorded at different time intervals. Data bars show mean±s.d. (*n* = 3 technical replicates).

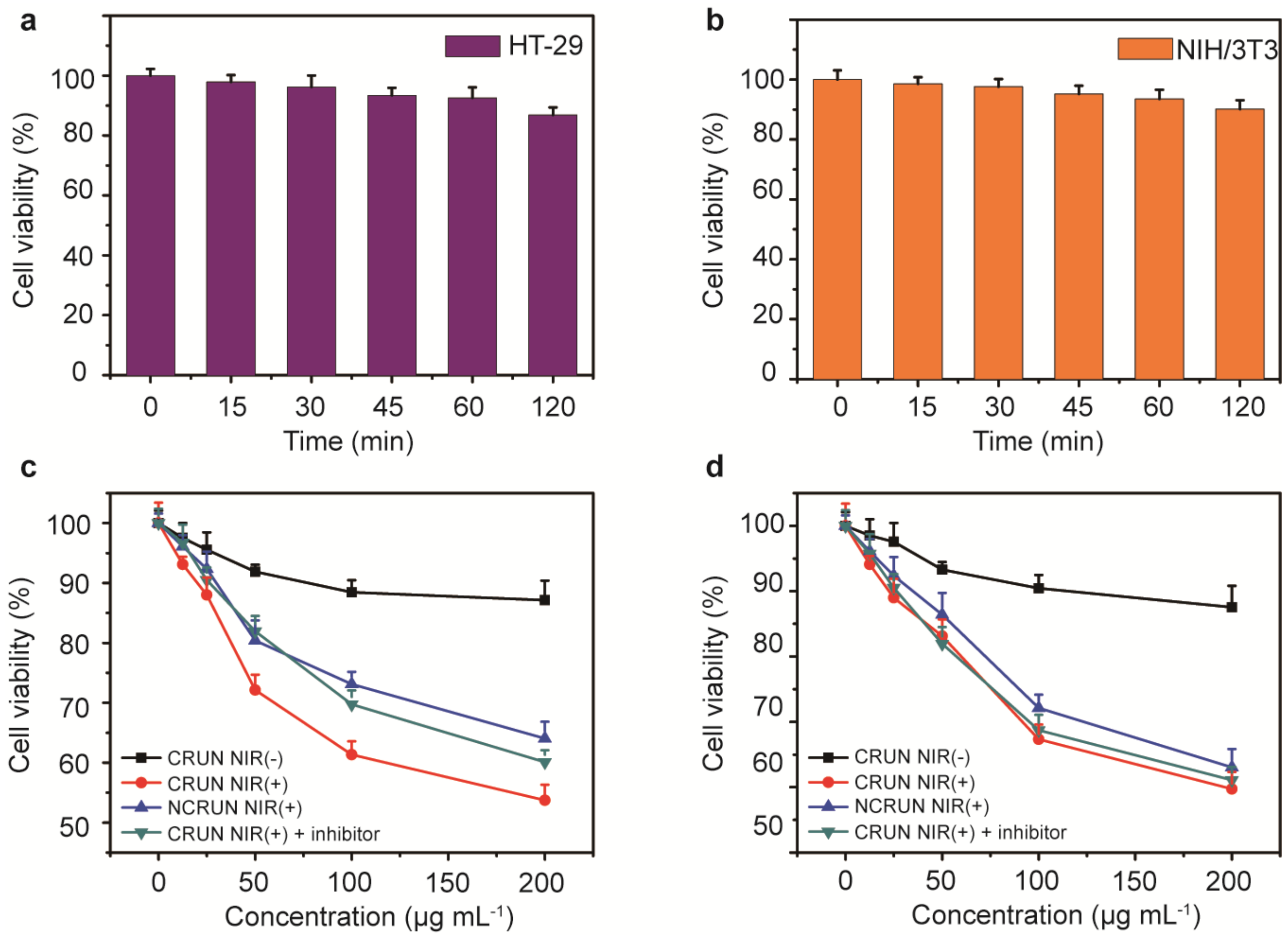

Supplementary Fig. 17. The photodynamic cytotoxicity assays of enzyme triggered CRUN in HT-29 and NIH/3T3 cells. (**a**) and (**b**) The phototoxicity of NIR light (at 808 nm) irradiation over increasing time on HT-29 (left) and NIH/3T3 (right) cells after incubating with CRUN (100 μg mL^{-1}) for 4 h. (**c**) and (**d**) The cell viability of HT-29 (left) and NIH/3T3 (right) cells incubated with different concentration of CRUN and the controlled non-cross-linking NCRUN with NIR light treatment (0.4 W cm^{-2}) for 1 h. The cells treated with CRUN but no light illumination and in the present of inhibitor (100 μM) were used as controls. Data bars show mean±s.d. (n = 3 technical replicates).

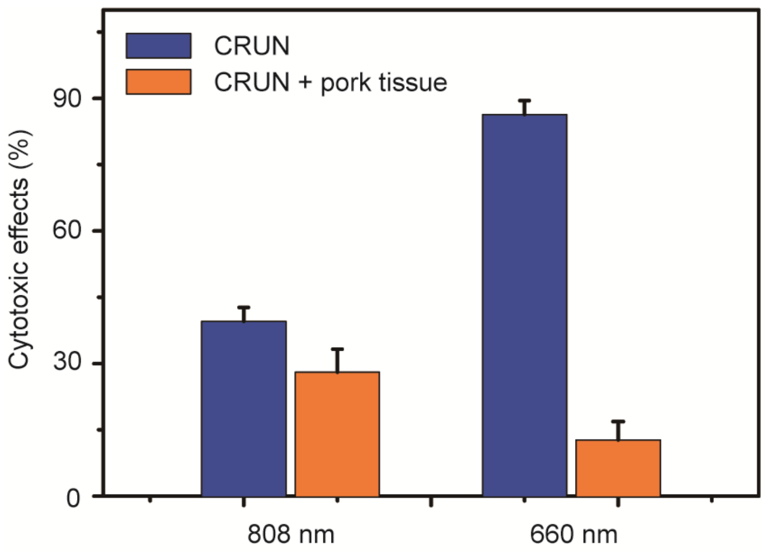

Supplementary Fig. 18. The photodynamic cytotoxicity assay of HT-29 cells incubated with CRUN in the absence and presence of 2-mm thick pork tissue upon 808 nm or 660 nm laser irradiation. Data bars show mean±s.d. (*n* = 3 technical replicates).

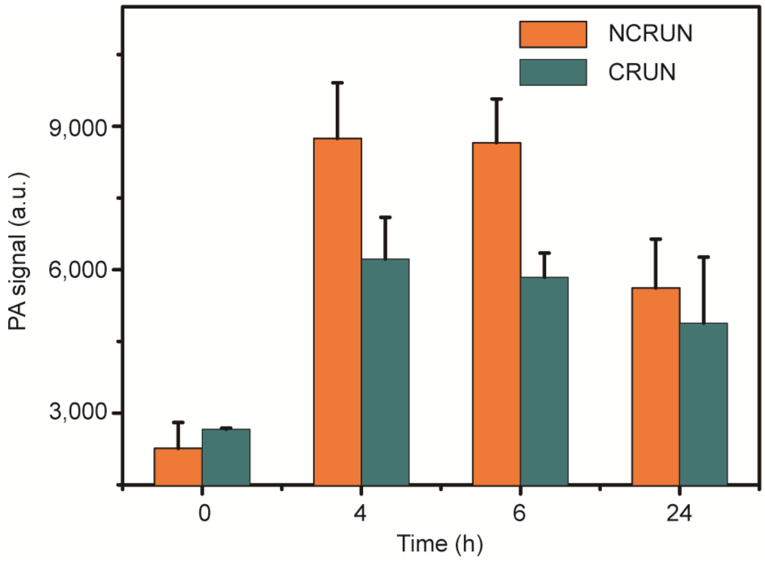

Supplementary Fig. 19. Living mice photoacoustic imaging upon intratumoral injection of CRUN. The PA signals were collected at different time after intratumoral injection of CRUN and control NCRUN (3 mg in 100 μL saline). Data bars show mean±s.d. (n = 3 technical replicates).

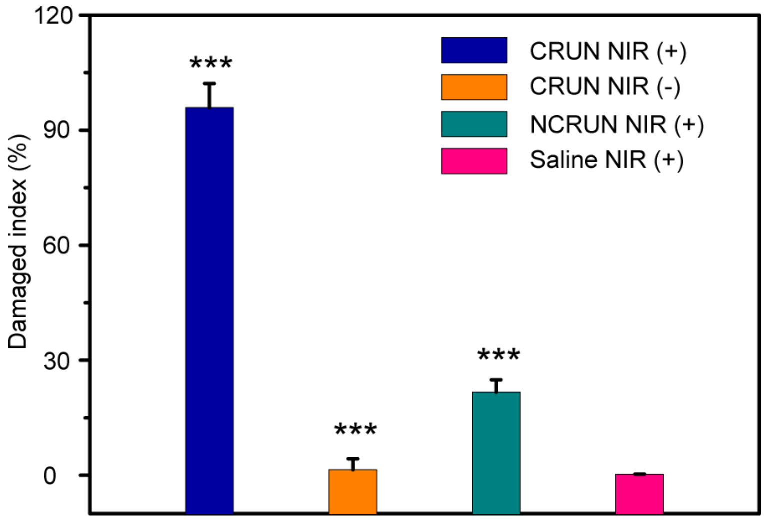

Supplementary Fig. 20. TUNEL staining of tumor tissue after intratumoral injection of CRUN. Data bars show mean±s.d. (n = 3 technical replicates), two-sided t-test, ***p < 0.001.

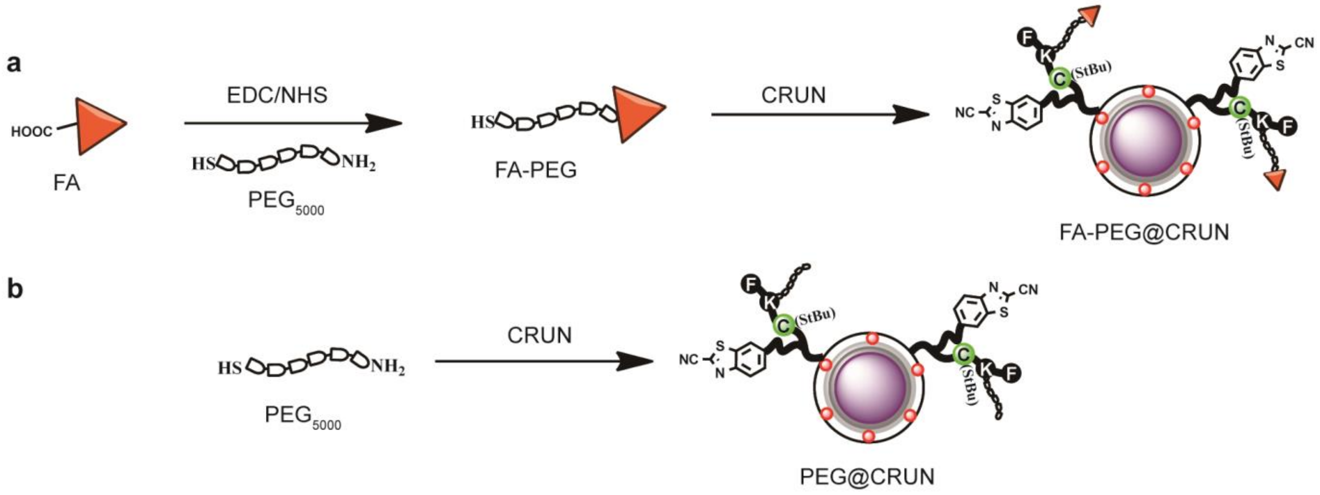

Supplementary Fig. 21. Illustration of synthetic scheme for FA-PEG@CRUN and PEG@CRUN.

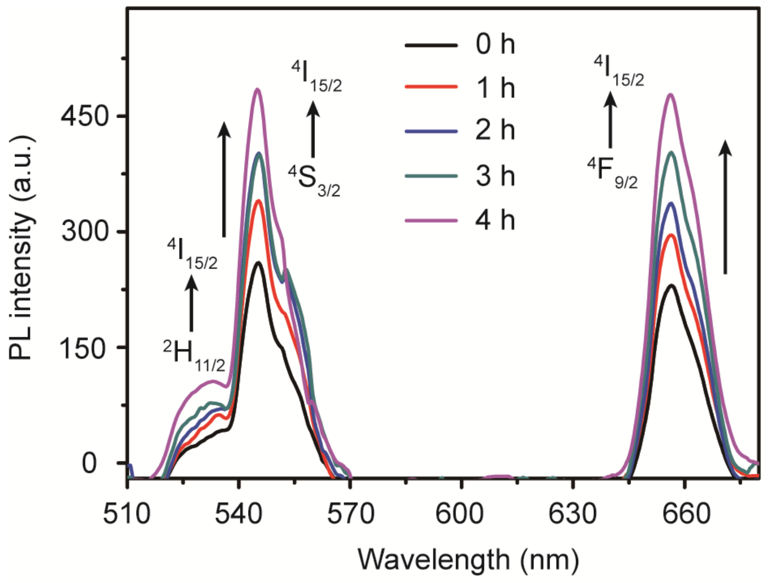

Supplementary Fig. 22. Luminescence analysis of FA-PEG@CRUN with CtsB treatment at different time. Fluorescence enhancement of CRUN (1.5 mg mL^{-1}) when treated with CtsB (55 nM) at different time intervals (0-4 h).

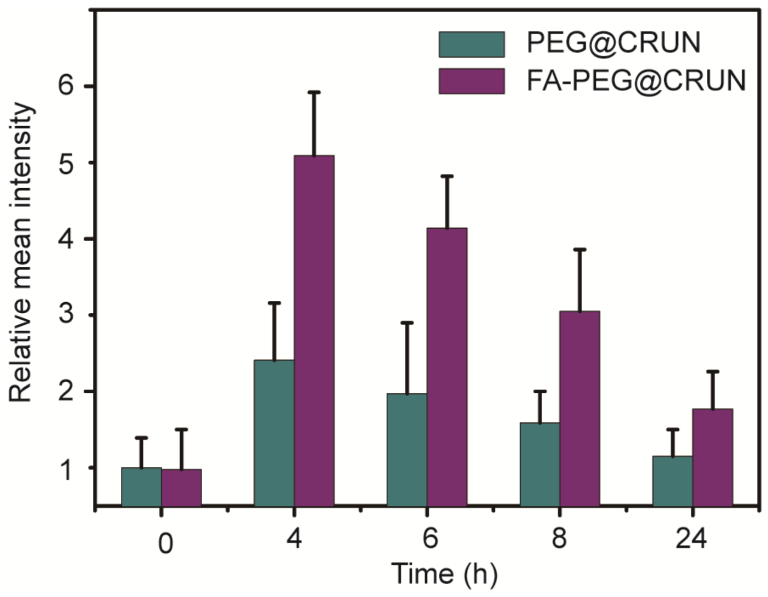

Supplementary Fig. 23. ***In vivo* quantification of fluorescence imaging signals in tumor region.** The prolonged fluorescence signals change was monitored after intravenous injection of FA-PEG@CRUN and PEG@CRUN (3 mg in 100 µl saline) in tumor bearing living mice. Data bars show mean±s.d. (*n* = 3 technical replicates).

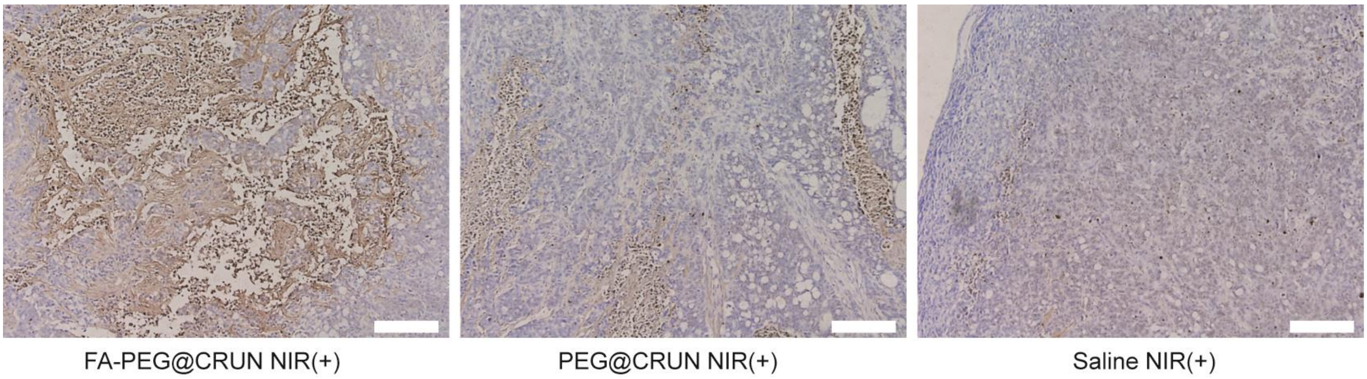

Supplementary Fig. 24. TUNEL histology of tumor tissues after NIR light mediated PDT treatment in tail vein injection of FA-PEG@CRUN and PEG@CRUN. Brown-stained cell nucleus represents damaged cells and blue-stained cell nucleus represents normal cells. Scale bar: 200 μm.

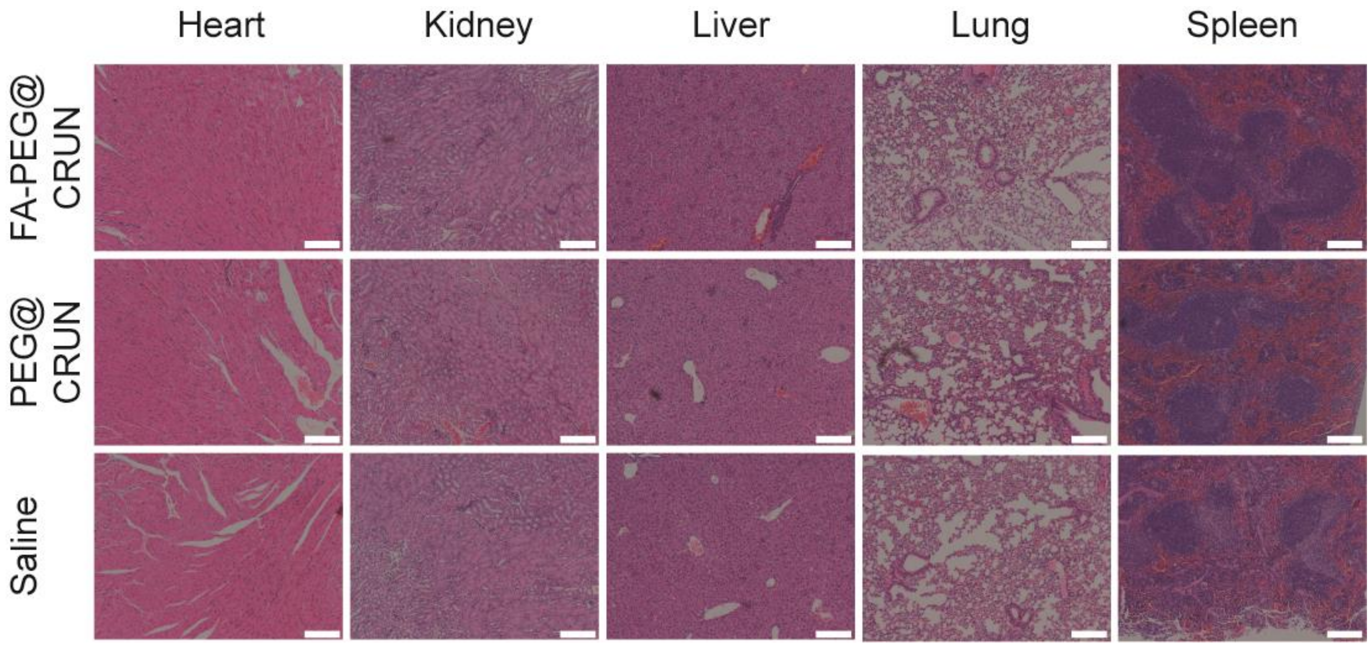

Supplementary Fig. 25. ***In vivo*** **H&E histology of various organ tissues.** Pathological H&E stained images of tissue sections from heart, liver, spleen, lung and kidney of the mice treated with FA-PEG@CRUN, PEG@CRUN and saline. The tumor tissue sections were harvested in 12 days after intravenous injection. Scale bar: 200 μm.

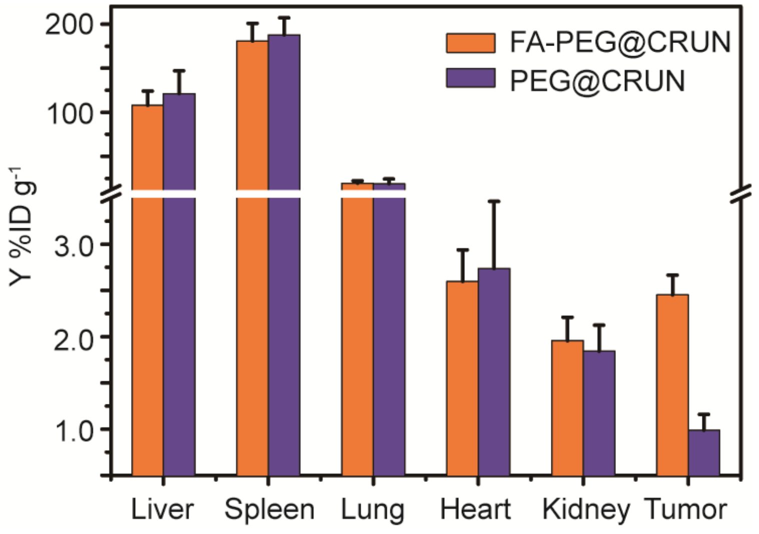

Supplementary Fig. 26. ICP analysis of bio-distribution of FA-PEG@CRUN and control PEG@CRUN in various organs. The living mice were sacrificed after 12 days PDT treatment with tail vein injection. The yttrium (Y^{3+}) concentration was analyzed by ICP-OES. Data bars show mean±s.d. (*n* = 3 technical replicates).

Supplementary Methods

Chemicals and reagents. $Y(CH_3CO_2)_3$, $Yb(CH_3CO_2)_3$, $Er(CH_3CO_2)_3$, $Nd(CH_3CO_2)_3$, oleic acid, 1-octadecene, NH_4F, NaOH, polyacrylic acid (PAA, Mw 1800), diethylene glycol (DEG), polyethylenimine (PEI, Mw 25000, branched), 1-Ethyl-3-(3-dimethylaminopropyl) carbodiimide hydrochloride (EDC·HCl), N-hydroxysuccinimide (NHS), 2-(1H-benzotriazol-1-yl)-1,1,3, 3-tetramethyluronium hexafluorophosphate (HBTU), diisopropylethylamine (DIPEA), isobutyl chloroformate (IBCF), 2-cyano-6-aminobenzothiazole (CBT), 4-methylmorpholine, folic acid (FA), tris(2-carboxyethyl) phosphine (TCEP), ethylenediaminetetraacetic acid (EDTA), *In vitro* toxicology assay kit (TOX8, resazurin based), cathepsin B (CtsB) from human liver, antipain hydrochloride inhibitor and 4',6-diamidino-2-phenylindole (DAPI) were purchased from Sigma-Aldrich. Chlorin e6 (Ce6) was purchased from Precision Technologies Pte Ltd (Singapore). Mal-dPEG$^{TM}{}_2$-NHS and NH_2-PEG_{5000}-SH (Mw 5000) were obtained from Quanta Biodesign and Laysan Bio, Inc., USA, respectively. Singlet oxygen sensor green (SOSG), Dulbecco's Modified Eagle Medium (DMEM), fetal bovine serum (FBS), penicillin-streptomycin and trypsin-EDTA were obtained from Invitrogen (Carlsbad, CA, USA). The solvents for synthesizing peptide were dehydrated and kept with activated 4 Å molecular sieves. All the commercially reagents were used as received unless otherwise noted.

Instruments. ^{1}H-NMR and ^{13}C-NMR spectra were measured on a 300 MHz Bruker spectrometer. High-resolution mass spectra were measured on a Waters Q-Tof Premier mass spectrometer (ESI measurements). Semi-preparative high performance liquid chromatography (HPLC, Shimadzu) system was performed on an Alltima C-18 column of 250×10 mm at a flow rate of 3 mL min^{-1}. UV-Vis absorption spectra were measured using a Beckman coulter DU800 spectrometer. Fluorescence emission spectra were captured with a Varian Cary eclipse fluorescence spectrophotometer. The fluorescence emission spectra of UCN was recorded at an angle of 90° to the excitation laser (808 nm) and an optical SEC-2000 spectrometer coupled 2048 pixels CCD assay (ALS Co., Ltd, Japan). A filter with short pass 750 nm was placed before the spectrometer to minimize scattering from the excitation laser beam. The luminescence lifetime decay curves and quantum yield were measured with a phosphorescence lifetime spectrometer (FSP920, Edinburgh) equipped with a microsecond flash lamp as the excitation source and an integrating sphere for efficiency data measurement. The resonance light scattering (RLS) spectra were recorded on a RF-5301PC spectrofluorophotometer (Shimadzu, Japan) at room temperature. Transmission electron microscope (TEM) images were recorded using a FEI EM208S TEM (Philips) operated at 100 kV. Dynamic light scattering (DLS) measurements were performed by Brookhaven 90 Plus Nanoparticle Size Analyzer. Yttrium element content was recorded using an Agilent 7700 Inductively Coupled Plasma Optical Emission Spectrometry (ICP-OES) in the present of standard yttrium nitric acid solution. The cell viabilities were measured by a Bio-Tek EL-311 microplate reader. Cell imaging was carried out on a confocal fluorescence microscope (Nikon, Eclipes TE2000-E) and upconversion imaging was also recorded on the same machine equipped with 980 nm laser wide-field fluorescence add-on (EINST Technology Pte Ltd, Singapore) in our lab. Photo-irradiation experiments were performed with a near-infrared (808 nm) and visible (660 nm) diode laser (Changchun New Industries Optoelectronics Technology Co., Ltd., China). All *in vitro* and *in vivo* photoacoustic (PA) imaging experiments were performed using a real-time multispectral optoacoustic tomographic (MSOT) imaging system (iThera Medical GmbH, Neuherberg, Germany). The animal fluorescence imaging was performed with an IVIS Lumina II imaging system (Caliper Life Sciences, Roissy, France).

1. Synthesis and characterization of CRUN and control NCRUN

The illustration of design and synthesis of enzyme triggered cross-linking CRUN and control NCRUN were shown in supplementary Fig. 1 with the following synthesis steps. The TEM and spectroscopic characterization of each step was shown in supplementary Fig. 2.

1.1) Synthesis of $NaYF_4$:Yb/Er/Nd (18/0.5/1%) core upconversion nanocrystals (UCNs):

The UCNs were obtained following a reference published before[1]. Typically, 2 mL methanol containing

$RE(CH_3CO_2)_3$ (RE = Y, Yb, Er and Nd), 3 mL oleic acid and 7 mL 1-octadecene were added in a 50 mL three-neck flask. The ratio of Yb/Er/Nd is 18/0.5/1% and the total lanthanide amount is 0.4 mmol. The mixture was heated to 150 °C for 60 min before cooling down to room temperature. Subsequently, a methanol solution (6 mL) containing NH_4F (59.3 mg) and NaOH (40.0 mg) was added and stirred for 30 min at 50 °C. Then methanol was evaporated and the solution was kept at 290 °C for 1.5 h under nitrogen atmosphere. After cooling down to room temperature, the core particles were precipitated using ethanol and collected through centrifugation several times after ethanol washing. Finally, the core particles were re-dispersed in 4 mL hexane.

1.2) Synthesis of $NaYF_4$:Yb/Er/Nd (18/0.5/1%)@$NaYF_4$:Nd (20%) core-shell UCNs:
Typically, 3 mL oleic acid and 7 mL 1-octadecene were added in a 50 mL three-neck flask, then 2 mL methanol containing 0.4 mmol $RE(CH_3CO_2)_3$ (RE = Y/Nd (80/20%)) was also added in the solution. The mixture was heated to 150 °C for 60 min before cooling down to room temperature. The as-synthesized $NaYF_4$:Yb/Er/Nd core particles in 4 mL hexane were added along with a methanol solution (6 mL) containing NH_4F (59.3 mg) and NaOH (40.0 mg). The resulting mixture was stirred for 30 min at 50 °C. Then methanol was evaporated and the solution was kept at 290 °C for 1.5 h under nitrogen atmosphere. After cooling down to room temperature, the core-shell particles were precipitated using ethanol and collected through centrifugation several times after ethanol washing. The absolute quantum yield of core-shell UCN was 0.22 % under excitation at 808 nm (80 W cm^{-2}) according to the standard process[2].

1.3) Synthesis of PAA@UCNs:
The PAA@UCNs synthesis was carried out following a literature protocol reported previously[3]. Briefly, 300 mg of PAA (1800 Da) and 30 mL DEG were added to a three-neck flask. The mixture was heated to 110 °C to form a clear solution. Then a hexane solution containing 100 mg core-shell UCNs was added slowly and the mixture was maintained at 110 °C for 1 h under nitrogen protection. After heating at 240 °C for 1.5 h, the resultant solution was cooled down to room temperature and precipitated with ethanol. The obtained PAA@UCNs was recovered by centrifugation (18000 rpm for 10 min) and washed three times with ethanol/water (1:1 v/v).

1.4) Synthesis of PEI/PAA@UCNs:
The PAA-capped UCNs were covalently encapsulated with branched PEI as reported previously[4]. Briefly, 30 mg of PAA@UCNs in 5 mL water were activated by EDC·HCl (50 mg) and NHS (5 mg) to form the active succinimidyl ester for 30 min. Then the mixture was added slowly to 10 mL PEI (2 mg mL^{-1}) in PBS (pH 7.4) with 30 min sonication. After 24 h magnetic stirring, the PEI/PAA@UCNs was purified by centrifugation (18000 rpm for 10 min) and washed three times with ethanol/water (1:1 v/v) to remove excess reactant.

1.5) Synthesis of enzyme responsive cross-linking peptide sequence and negative control peptide:
All peptide sequences were synthesized by using standard Fmoc-chemistry based solid phase peptide synthesis (SPPS) with 2-Chlorotrityl Chloride Resin. HBTU/DIPEA was used for coupling reaction at room temperature and 20% piperidine/DMF solution was used to deprotect Fmoc group in the terminal of peptide. The synthetic route of enzyme responsive cross-linking peptide sequence Ac-FKC(StBu)AC(SH)-CBT was shown in supplementary Fig. 3 as the following process[5].

1.5.1) Synthesis of substrate 1:
The isobutyl chloroformate (IBCF, 23.4 mg, 0.17 mmol) was added the mixture of Fmoc-Cys(Trt)-OH (72.9 mg, 0.12 mmol) and 4-methylmorpholine (MMP, 34.6 mg, 0.34 mmol) in 3 mL anhydrous THF at 0 °C under N_2 atmosphere and the mixture was stirred for 20 min. Then 2-cyano-6-aminobenzothiazole (CBT, 20.0 mg, 0.11 mmol) was added to the mixture for further stirring at 0 °C for 2 h before overnight at room temperature. After evaporating the THF solvent under reduced pressure, 10 mL water was added to the reaction mixture with extracting with 10 mL ethyl acetate for three times. The organic phase was combined and dried using Na_2SO_4. The mixture was concentrated under reduced pressure and subsequently a flash column chromatography with

eluent ethyl acetate and hexane is performed to obtain a white product (53.1 mg, 65%). ^{1}H NMR (300 MHz, $(CD_3)_2SO$): δ8.74 (s, 1H), 8.21 (d, 1H, *J* = 6Hz), 7.97-7.85 (m, 6H), 7.38-7.25 (m, 19H), 4.44 (d, 1H, *J* =3Hz), 4.34-4.25 (m, 3H), 3.38 (s, 2H), 3.20 (d, *J* = 3Hz, 1H). ^{13}C NMR (75 MHz, $(CD_3)_2SO$): 170.0, 156.2, 148.3, 144.6, 144.2, 144.1, 141.2, 139.5, 137.1, 135.7, 129.6, 128.6, 128.3, 128.1, 127.5, 127.3,125.8, 125.3, 121.4, 120.5, 114.0, 112.2, 66.6, 66.4, 55.1, 49.1, 47.1, 34.3; HRMS (m/z): calcd. for $C_{45}H_{34}N_4O_3S_2Na^+$ $[M+Na]^+$: 765.1970, found 765.1966.

1.5.2) Synthesis substrate 2:

Substrate **1** (40.0 mg, 0.05 mmol) of was added into 5 % piperidine dissolved in 3 ml of DMF and allowed to stir under ambient temperature for 20 min. Next, 5 mL of water was added to the reaction mixture and extracted with 10 mL ethyl acetate for three times. The organic phase was combined and dried using Na_2SO_4. After concentrating the mixture under reduced pressure, the sample was purified using flash chromatography with choice of eluent as ethyl acetate and petroleum ether to obtain substrate **2** (19 mg, 73%). ^{1}H NMR (300 MHz, $(CD_3)_2SO$): δ 8.76 (s, 1H), 8.18 (d, 1H, *J* = 6Hz), 7.79 (dd, 1H, *J* = 6Hz, 3Hz), 7.35 (m, 16H), 3.42 (t, 1H, *J* = 6Hz), 3.31 (s, 2H). ^{13}C NMR (75 MHz, $(CD_3)_2SO$): 173.2, 148.2, 144.9, 139.7, 137.2, 135.5, 129.6, 128.5, 127.2, 125.2, 121.4, 114.0, 111.8, 66.4, 55.5, 37.3; HRMS (m/z): calcd. for $C_{30}H_{24}N_4OS_2Na^+$ $[M+Na]^+$: 543.1289, found 543.1293.

1.5.3) Synthesis substrate 3 and enzyme responsive cross-linking peptide of Ac-FKC(StBu)AC(SH)-CBT:

Substrate **2** (17.3 mg, 0.033 mmol) was dissolved in 1 mL of DMF solution containing Ac-Fk(Boc)C(StBu)A-OH peptide (28.9 mg, 0.041 mmol). EDC (10.3 mg, 0.066 mmol) of and NHS (10.1 mg, 0.066 mmol) were then added into the reaction mixture. Finally, DIPEA (14.5 µl, 0.083 mmol) was added into the reaction flask and allowed to stir over night under ambient condition. After monitoring the starting materials has been consumed in the reaction through TLC, the reaction mixture was extracted using 10 ml of ethyl acetate, water and brine and finally dried over anhydrous sodium sulphate. After the reaction mixture has been concentrated under reduced pressure. The crude mixture of **3** obtained was dissolved in 1ml of DCM and subsequently trifluoroacetic acid (TFA) and triisopropylsilane (TIPS) were added into the reaction flask. The mixture was then purified using reverse phase HPLC to obtain enzyme responsive cross-linking peptide (28.3 mg, 42% in two steps). ^{1}H NMR (300 MHz, $(CD_3)_2SO$): δ 8.74 (s, 1H), 8.21 (d, *J* =3Hz, 4H), 8.18 (d, *J* =3Hz, 2H), 7.80-7.73 (m, 4H), 7.25-7.16 (m, 6H), 4.56-4.54 (m, 3H), 4.34-4.29 (m, 2H), 3.16-3.12 (m, 2H), 2.98-2.87 (m, 8H), 1.75 (s, 3H), 1.55 -1.47 (m, 4H), 1.26 (s, 9H), 1.22 (s, 3H); ^{13}C NMR (75 MHz, $(CD_3)_2SO$):172.6, 172.1, 169.9, 169.6, 148.3, 139.6, 138.5, 137.1, 135.4, 129.6, 128.5, 126.7, 125.3, 121.4, 114.0, 112.0, 56.5, 54.5, 53.0, 52.8, 49.0, 48.2, 42.9, 37.7, 31.7, 30.0, 29.9, 27.0, 26.3, 22.9, 22.6, 18.3; HRMS (m/z): calcd. for $C_{38}H_{52}N_9O_6S_4$ $[M+H]^+$: 858.2923, found: 858.2914.

1.5.4) Synthesis of control non-cross-linking peptide:

The control peptide sequence Ac-FKC(StBu)AC without cross-linking property was obtained using the conventional solid phase peptide synthesis (SPPS) with 2-Chlorotrityl Chloride Resin. HBTU/DIPEA was used for coupling reaction at room temperature and 20% piperidine / DMF solution was used to deprotect Fmoc group in the terminal of peptide. After washing with DMF and DCM several times, 95% TFA was used to cleave the peptide from the resin and remove Boc and Trt protecting group under stirring for 2 h. The negative control peptide was purified using reverse-phase HPLC and then frozen-dried with lyophilizer. ^{1}H NMR (300 MHz, $(CD_3)_2SO$): δ 8.20 (s, 2H), 8.10 (t, *J* = 6Hz, 3H), 7.80 (s, 3H), 7.26 (s, 4H), 7.20-7.18 (m, 1H), 4.52-4.40 (m, 2H), 4.35-4.29 (m, 3H), 3.43-3.37 (m, 3H), 3.17-3.98 (m, 5H), 2.39 (t, *J* = 9Hz, 1H), 1.77 (s, 3H), 1.70 (s, 1H), 1.57-1.55 (m, 3H), 1.29 (s, 9H), 1.24 (s, 3H), 1.00-0.99 (m, 1H). ^{13}C NMR (75 MHz, $(CD_3)_2SO$): δ172.3, 172.0, 171.9, 171.8, 169.9, 169.6, 138.5, 129.6, 128.5, 126.6, 54.7, 54.5, 53.0, 52.7, 48.6, 48.1, 42.9, 42.6, 37.8, 31.7, 31.0, 30.0, 27.1, 26.0, 22.9, 22.6, 18.5, 18.3, 12.5; HRMS (m/z): calcd. for $C_{30}H_{48}N_6O_7S_3[M+H]^+$: 701.2785, found 701.2780.

1.6) Synthesis of enzyme responsive cross-linking of rare-earth upconversion nanostructures (CRUN):
Firstly, Ce6 (14.4 mg) was activated with EDC (23.0 mg) and NHS (13.8 mg) for 30 min. Then PEI/PAA@UCNs (20.0 mg) was dissolved in PBS (10 mM, pH=7.4) with sonication containing Mal-dPEG$^{TM}{}_2$-NHS (10.0 mg) ester linker and active Ce6-NHS ester. After stirring for 24 h, the mixture was precipitated by centrifugation and washed with water thrice, and then it was dissolved in 10 mL PBS (10 mM, pH=7.4) containing enzyme responsive cross-linking peptide sequence Ac-FKC(StBu)AC(SH)-CBT (20.6 mg) in 200 μL DMSO. Accordingly, the controlled non-cross-linking particle conjugate (NCRUN) was prepared following the same protocol with Ac-FKC(StBu)AC. The final products CRUN and NCRUN were obtained by centrifugation and washed with water three times. The successful formation of enzyme responsive cross-linking peptide and Ce6 modified UCNs conjugate (CRUN) was confirmed by UV-Vis spectroscopic characterization (Supplementary Fig. 4). The loading percentage of Ce6 on UCNs was determined by UV-Vis absorbance peak at 665 nm which is comparable other reported values[3,6]. The amount of enzyme responsive cross-linking peptide on UCN was determined by monitoring the fluorescence emission of CBT group (λ_{ex}= 320 nm, λ_{em}= 450 nm) as shown in supplementary Fig. 5.

2. *In vitro* enzyme triggered covalent cross-linking of CRUN

2.1) Enzyme triggered cross-linking reaction of CRUN in buffer:
The reaction scheme of enzyme triggered cross-linking was shown in supplementary Fig. 6. Briefly, the stock solution of CtsB protease enzyme was dissolved in 25 mM of acetate buffer solution (pH 5.0) with 1 mM EDTA. The upconverted luminescence (UCL) spectrum in quartz cuvette was recorded after incubating CtsB (55 nM) and CRUN (1.5 mg mL^{-1}) in working buffer (0.1 M sodium phosphate, 0.05 M NaCl, 1mM EDTA, pH 6.0) at 37 °C containing TCEP (5 mM, a commonly used reductive agent) for different time[7]. In the negative control experiment, the typical CtsB inhibitor[8], antipain hydrochloride (100 μM), was pretreated with CtsB for 2 h at 37 °C and then incubated with CRUN for different time as above. After enzyme triggered cross-linking reaction, the CRUN were separated by centrifugation and re-dispersed in ethanol for TEM test (supplementary Fig. 7).

The UV-Vis absorption and luminescence spectra of CRUN in the presence of CtsB and anitipain inhibitor were measured after 4 hours incubation. In order to confirm the possibility of the cross-linking reaction, the formation of the covalent cross-linking product, firefly luciferin, was monitored by the fluorescence change at 489 nm (320 nm light as excitation wavelength)[5] (supplementary Fig. 8).

In the mean time, the lanthanide UCL of CRUN with CtsB treatment was also measured with 808 nm excitation at different time intervals (supplementary Fig. 9). The luminescence photographs of different CRUN samples in the absence of CtsB, in the presence of CtsB, or with both CtsB enzyme and inhibitor treatment were recorded in the dark, respectively. The relevant fluorescence intensity was measured by Image J software (supplementary Fig. 10). The luminescence decay curves of lanthanide dopants (Er^{3+}, Nd^{3+} and Yb^{3+}) in CRUN were measured in the absence and presence of CtsB enzyme after 4 h incubation (supplementary Fig. 11). After enzyme triggered cross-linking reaction (4 h), the CRUN were separated by centrifugation and the precipitates were re-dispersed in 0.1 mL DMSO solution. The resonance light scattering (RLS) spectra were obtained by scanning synchronously with the same excitation and emission wavelength ($\lambda_{ex} = \lambda_{em}$) from 808 to 900 nm (supplementary Fig. 12)[9]. Both the excitation and emission slit widths were kept at 10.0 nm.

2.2) Quantification of cathepsin B and folate receptor expression in HT-29 and NIH/3T3 cells:
The human colorectal adenocarcinoma cell line (HT-29, cat. no. HTB-38) and mouse embryonic fibroblast cell line (NIH/3T3, cat. no. CRL-1658) were provided from American-type culture collection (ATCC) and checked for mycoplasma contamination. These cells are not listed by ICLAC as misidentified cell lines (October 3rd, 2014). Both these two cells were cultured in high-glucose DMEM containing 10% FBS, penicillin (100 I.U. mL^{-1}) and streptomycin (100 μg mL^{-1}) at 37 °C in a humidified atmosphere with 5% CO_2. The quantification of

Cathepsin B (CtsB) in HT-29 cells (40.1±4.4 ng mg^{-1} protein) and NIH/3T3 cells (2.2±1.4 ng mg^{-1} protein) were performed by Human Cathepsin B ELISA (Enzyme-linked immunosorbent Assay) kit according to the procedure described by the manufacturer (CUSABIO, USA)[10]. The protein content was determined by using bicinchoninic acid (BCA) method after protein extraction from 1×10^5 cells based on the standard protocol (Thermo Scientific, USA)[11]. The quantification of folate receptor levels in HT-29 cells ($3.8\pm0.3\times10^5$ folate receptors per cell) was also performed by Human FOLR1/Folate Receptor Alpha ELISA kit according to the procedure described by the manufacturer (CUSABIO, USA)[12].

Western blot assay was also performed to compare the difference expression levels of CtsB in these two cell lines (Supplementary Fig. 13). Briefly, 1.2×10^6 HT-29 or NIH/3T3 cells were lysed with 50 μL 2×SDS sample buffer and boiled at 90˚C for 5 min. Then 20 μL of each samples were analyzed by 10% SDS-PAGE and transferred to 0.22 μm PVDF membrane (100 V, 110 min, 4 ˚C). The membrane was blocked with 5% milk (PBST) for 1h, and incubated with rabbit anti-Cathepsin (ab125067, Abcam) antibody (1:750 dilutions) at 4 ˚C overnight. The membrane was washed 3 times with PBST, then incubated with goat anti-rabbit 2nd antibody (1: 10000 dilutions) for 1h at RT and washed 3 times with PBST. The samples were detected with LAS-4000 machine using enhanced chemiluminescence (ECL) exposure buffer. After that, another 20 μL (the same amount as the above) of each samples were analyzed in 10% SDS-PAGE and transferred to 0.22 μm PVDF membrane (100 V, 110 min, 4˚C). The membrane was incubated with rabbit anti-GAPDH antibody (sc-25778, Santa Cruz, 1:1000 dilutions) for 1 h at RT, washed 3 times with PBST, then incubated with goat anti-mouse 2nd antibody (1: 10000 dilutions) and revealed by ECL.

2.3) Fluorescence imaging of enzyme triggered particle cross-linking in living cells:

Cathepsin B (CstB) over-expressing HT-29 cells and cathepsin B deficient NIH/3T3 cells were seeded with a cell density of 1×10^5 in an ibidi confocal μ-dish (35 mm, plastic bottom) in 1 mL DMEM media which was incubated for 24 h at 37 °C. The seeded cells were subsequently treated with CRUN (50 μg mL^{-1}) and incubated for 4 h. The cell nuclei were stained by DAPI (1 μg mL^{-1}) for 20 min and then the cells were washed with PBS (pH 7.4) for three times. As controls, the HT-29 cells and NIH/3T3 cells were treated with CRUN in the present of CtsB inhibitor. These two cells were also treated with non-cross linking NCRUN conjugates, which were used as another control. For the cell inhibition assay, the cells were first pretreated with CtsB inhibitor (antipain hydrochloride, 100 μM in DMEM) for 2 h at 37 °C follow by the addition of CRUN and incubated for a further 4 h. Fluorescent imaging of the samples were then carried out under confocal microscopy system with a continuous-wave 980nm laser as excitation wavelength (EINST Technology Pte Ltd). The cellular imaging data of enzyme triggered CRUN cross-linking in HT-29 and NIH/3T3 cells were shown in Fig. 2g and supplementary Fig. 14. The fluorescence analysis of enzyme triggered particle cross-linking in these two cells were measured by Image J (supplementary Fig. 15).

2.4) Singlet oxygen analysis in buffer solution:

Singlet oxygen ($^1O_2$) was detected based on a highly sensitive probe singlet oxygen sensor green (SOSG) according to the protocol reported previously[13] (Fig. 2i and Supplementary Fig. 16). Briefly, SOSG (100 μg per tube) was dissolved in 33 μL methanol as stock solution (5 mM). Different samples containing Ce6 (1 μM) were mixed with SOSG (2.5 μM) in PBS (10 mM, pH 7.4) and then irradiated by an 808 nm or 660 nm laser (0.4 W cm^{-2}) for different periods of time in the dark.

In addition, a 2-mm thick pork tissue (adipose tissue) was used to mimic living condition for further investigation of the different penetration between 808 nm (NIR) and 660 nm light irradiation (Fig. 2i). The generation of singlet oxygen was based on the enhanced fluorescence of SOSG at 530 nm compared with background signals under the excitation wavelength at 494 nm.

2.5) *In vitro* photodynamic cytotoxicity assay of CRUN in HT-29 and NIH/3T3 cells:

The HT-29 and NIH/3T3 cell lines were seeded in a 96-well plate (1×10^4 cells per well in 100 μL DMEM) and cultured for 24 hours at 37 °C. The cells were incubated with CRUN at various concentrations for 4 h and then washed with fresh medium to remove free nanoparticles. For *in vitro* PDT treatment, cells were exposed to 808 nm or 660 nm irradiation at the same power density of 0.4 W cm^{-2} for 60 min in the dark (10 min break after 10 min irradiation). Similar control experiments were employed as following: cells treated with CRUN in the absence of laser illumination; with CRUN in the presence of CtsB inhibitor (antipain hydrochloride, 100 μM); with controlled NCRUN conjugates in the presence of laser irradiation; and cells treatment by NIR light irradiation only but without particle incubation.

Moreover, to demonstrate the deep-tissue penetration advantage of 808 nm (NIR) light for PDT treatment, CRUN was incubated with enzyme over-expressed HT-29 cells and treated with 660 nm or 808 nm light irradiation in the absence or presence of 2 mm thick pork tissue for 1 h under the same optical power density. After PDT treatment, the cells were incubated for another 48 h in the dark. The cell viabilities were evaluated by *in vitro* toxicology assay kit (TOX8, resazurin based) according to standard manufacturer's protocol[14]. Each experiment was repeated three times and the average values were used for analysis (supplementary Fig. 17 and 18).

2.6) Photoacoustic (PA) imaging experimental protocol and parameters:

Phantom *in vitro* and *in vivo* photoacoustic (PA) imaging experiments were performed using MSOT imaging system[15]. Typically, the optical excitation was provided by an optical parametric oscillator (OPO) with a tunable NIR wavelength which ranges from 680 nm to 980 nm that is in turn pumped by a Q-switched Nd:YAG laser with an average pulse duration of about 10 ns and repetition rate of 10 Hz. The PA signals were acquired using a 128-element concave transducer array spanning a circular arc of 270°. This transducer array has a central frequency of 5 MHz, which is used to provide a transverse spatial resolution in the range of 150-200 mm. During the process of image acquisition, the sample is being translated *via* the transducer array along its axis across the volume region of interest (ROI). In the process of *in vivo* imaging, the ultrasound gel was applied on the surface of the mouse skin and subsequent measurements were acquired in temperature-controlled water for a good acoustic coupling. For the PA experiments of i.v. injection, a similar PA imaging experiment was conducted under the same condition of acoustic signals were acquired using a 256-element tomographic ultrasound detector array spanning a circular arc of 270° with a central frequency of 5 MHz in the same system whole body imaging were operated under conditions of 0.3 mm step distance along the long axis of animal and 10 repeat pulse per position in designed time point. PA signal intensity of tumor was measured with region of interest (ROI) assay. Considering the multiple absorption peaks of lanthanide doped upconversion nanostructures due to their specific f-f transitions[16], in our study, the multi-wavelength PA signals were recorded from 680 nm to 980 nm illumination, and the maximum contrasted PA signal can be obtained when the excitation wavelength is 680 nm during PA imaging experiments.

2.7) *In vitro* PA imaging of enzyme triggered particle cross-linking in buffer solution:

The stock solution of CRUN (1.5 mg mL^{-1}) and CtsB (55 nM) were first incubated in 200 μL working buffer (0.1 M sodium phosphate, 0.05 M NaCl, 1 mM EDTA, pH 6.0) at 37 °C containing TCEP (5 mM) as reduction agent for different time. Then the solution was loaded into a PA phantom containing two-channel polyurethane cylindrical for PA testing, one for holding the control medium (working buffer) and the other for holding CRUN after incubation with enzyme for different time. The PA signal was recorded using a 128-element concave transducer array spanning a circular arc of 270° with the optimal excitation wavelength at 680 nm. In the negative control experiment, a typical inhibitor, antipain hydrochloride (100 μM) was first pretreated with CtsB for 2 h at 37 °C and then incubated with CRUN for different time as same protocol.

2.8) *In vitro* PA imaging of enzyme triggered particle cross-linking in living cells:

HT-29 cells and NIH/3T3 cells were cultured in a 65 mm dish with a density of 4 million cells per well in 2 mL

DMEM media and incubated for 24 h at 37 °C. The cells were incubated with CRUN (1.5 mg mL^{-1}) for 4 h at 37 °C incubator. In the control experiment, the cells were pretreated with CtsB inhibitor (antipain hydrochloride, 100 μM in DMEM) for 2 h at 37 °C before the addition of CRUN for 4 h incubation. Then cells were harvested using trypsin and washed three times with PBS. The cells were re-dispersed in 0.2 mL PBS for PA imaging test in two-channel cylindrical.

3. *In vivo* enzyme triggered covalent cross-linking of CRUN

3.1) *In vivo* animal studies:

All animal experimental procedures were performed in accordance with the protocol #120774 approved by the Institutional Animal Care and Use Committee (IACUC). Female Balb/c nude mice (~6-8 week old) were purchased from Charles River Laboratories (Shanghai, China). Xenograft mice models were established by injecting 0.1 mL of tumor cells suspension (PSB/matrigel, BD biosciences 1:1, v/v) containing 3×10^6 HT-29 into both flanks or only right side of mouse. When the tumor volume reached a palpable size, the mouse was used for further studies.

3.2) *In vivo* PDT treatment through intratumoral injection of enzyme triggered cross-linking of CRUN in tumor bearing mice:

Xenograft mice models were treated when the tumor volumes approached to 3-5 mm in diameter. For *in vivo* PDT treatment, the tumor-bearing mice were randomized into four groups (n = 8, each group) and treated by intratumoral injection of particle samples. In group 1, CRUN (3 mg in 100 µL saline) was directly injected into implanted tumors, followed by an 808 nm laser irradiation after 4 h injection. In group 2, same amounts of controlled NCRUN conjugate was injected with subsequent laser exposure. Control experiments with injection of CRUN but no NIR light irradiation (group 3) and saline with laser treatment (group 4) were employed, respectively. The effective exposure time for each mouse was 45 min (5 min interval after 5 min irradiation with total treatment time of 90 min) with power density at 0.4 W cm^{-2} (fluence 1080 J cm^{-2}). A second and third dose injection followed with PDT treatment described above was repeated at 4 d and 8 d after the first dose injection, respectively. Tumor size was measured three times a week using a vernier caliper upon PDT treatment. The tumor volume was calculated using the following equation: tumor volume (V) = length × width^2/2. Relative tumor volume was calculated as V/V_0 (V_0 was the initial tumor volume before PDT treatment).

3.3) *In vivo* PA imaging of enzyme triggered cross-linking in tumor region:

For *in vivo* PA imaging of intratumoral injection of CRUN and NCRUN (3 mg in 100 µL saline), the tumor bearing mice was anaesthetized with 3% isoflurane and ultrasound gel was applied on the surface of the mouse skin. Next, the distribution of the probe in tumor region was monitored over a period of time using PA imaging. In order to obtain an image acquisition, several slices with a step size of about 0.3 mm spanning from the liver all the way to abdomen was manually selected by live MSOT images with 8 laser excitation wavelengths from 680 nm to 980 nm (50 nm interval). Multispectral imaging was then performed with 10 signal averages per wavelength per transverse slice before injection and 4, 8 and 24 h post-injection (supplementary Fig. 19).

3.4) Histology examination of tumor tissues after treatment:

Mice were sacrificed after the multiple intratumoral injections with NIR light mediated PDT treatment. The tumor tissues were collected and fixed with 4% paraformaldehyde solution, processed routinely into paraffin and sectioned into 8 microns thick slices. After stained with hematoxylin & eosin (H&E) followed by standard protocol[17,18], the slices were examined by a digital optical microscope (BX 51, Olympus, USA). Damaged cells in tumor region was performed using the terminal deoxyribonucleotide transferase-mediated nick-end labeling (TUNEL) assay using an *in situ* damaged cell detection kit (Roche, Basel, Switzerland) according to the manufacturer's instructions[19]. The extent of damaged cells was evaluated by counting the number of TUNEL-positive (brown-stained) cells. The damaged index was calculated as the number of TUNEL-positive

cells divided by the total number of cells in 10 randomly selected high-power fields, as shown in supplementary Fig. 20.

3.5) Synthesis of tumor affinity ligand conjugated CRUN (FA-PEG@CRUN):

The illustration of synthetic scheme of FA-PEG@CRUN and PEG@CRUN were shown in supplementary Fig. 21. First, the FA-PEG_{5000}-SH conjugate was synthesized according to previous study[20,21]. Briefly, FA (44.2 mg) was activated with EDC (48.0 mg) and NHS (28.8 mg) in DMSO (6 mL) under N_2 for 30 min. The resulting activated FA solution was added dropwise to the DMSO solution (2 mL) of NH_2-PEG_{5000}-SH (Mw 5000, 100 mg) with triethylamine (TEA, 145.7 μL) under vigorous magnetic stirring in the dark at room temperature for 24 h. Then the reaction mixture was dialyzed in water using a dialysis membrane with molecular weight cut-off (MWCO) of 3500 Da for 3 days and followed by lyophilization to obtain the product FA-PEG_{5000}-SH. Second, the as-prepared CRUN (40.0 mg) was activated by Mal-$dPEG^{TM}_2$-NHS (20.0 mg) ester linker in PBS (10 mM, pH = 7.4) for 12 h. After remove the excess reactants, the Mal-activated UCN complex was re-dissolved in PBS (10 mM, pH = 7.4) and then FA-PEG_{5000}-SH (25.0 mg) was added into the solution with vigorous magnetic stirring in the dark at room temperature for 24 h. Accordingly, the PEG modified CRUN particles but without FA as affinity ligand (PEG@CRUN) were prepared as controlled conjugate following the same protocols with NH_2-PEG_{5000}-SH (25.0 mg) modification. The final products FA-PEG@CRUN and PEG@CRUN were obtained by centrifugation and washed with water for three times. The luminescence analysis of FA-PEG@CRUN (1.5 mg mL^{-1}) at different time intervals after CtsB enzyme treatment (55 nM) was followed the same protocol as previous CRUN platform without FA modification (supplementary Fig. 22).

3.6) *In vivo* targeted PDT treatment with intravenous injection of enzyme triggered FA-PEG@CRUN:

Xenograft HT-29 tumor model in the right side of female Balb/c mice were first developed as described above. For *in vivo* tumor targeting PDT treatment, the tumor-bearing mice have been randomly arranged into six different groups (n = 8, each group) to perform a series of intravenous injection of saline (group 1), PEG@CRUN (group 2) and FA-PEG@CRUN (group 3-6) respectively (3 mg in 100 μl saline). After 4 h of i.v. injection, laser treatment was performed on groups 1-6 by irradiation the tumor region with a continuous 808 nm or 660 nm laser. The effective exposure time for each mouse was 45 min (5 min interval after 5 min irradiation with total treatment time of 90 min) with power density at 0.4 W cm^{-2} (fluence 1080 J cm^{-2}). The tumor in group 4 and 6 were covered by 2 mm thick pork tissue and then irradiated with 808 nm or 660 nm lights under the same condition[22,23]. The PDT treatment with laser irradiation was repeated every two days. A second and third dose injection of the PDT treatment described above was repeated 4 d and 8 d after the first dose injection, respectively. Tumor size was measured and calculated as described above.

3.7) *In vivo* fluorescence imaging in living mice:

For *in vivo* luminescence imaging of live animals, the stock solution of PEG@CRUN and FA-PEG@CRUN were prepared by dissolving the substrates in saline (3 mg in 100 μl saline). The tumor bearing mice was anaesthetized with isoflurane and imaged immediately after tail vein injection at the indicated time points of 0, 4, 8 and 24 h post-injection. The optical imaging of Ce6 molecules (λ_{em} = 670 nm) loading at UCNs were collected using an IVIS Lumina II imaging system (Caliper Life Sciences, Roissy, France) with the excitation wavelength of 640 nm and analyzed with the living Image 2.11 software package. For quantification of measured signals, regions of interest were drawn over the tumor region with blue circle and final values were expressed by averaging the obtained signals from the all the mice scanned in each experiment (supplementary Fig. 23).

3.8) *In vivo* PA imaging of enzyme triggered cross-linking in tumor region:

For *in vivo* PA imaging of intravenous injection of FA-PEG@CRUN and PEG@CRUN (3 mg in 100 μL saline), the tumor bearing mice was anaesthetized with 3% isoflurane and ultrasound gel was applied on the surface of the mouse skin. The location of the probe in tumor region was monitored over a period of time using PA imaging. In order to obtain an image acquisition, several slices with a step size of about 0.3 mm spanning from the liver all

the way to abdomen was manually selected by live MSOT images with 8 laser excitation wavelengths from 680 nm to 980 nm (50 nm interval). PA imaging was performed with 10 signal averages per wavelength per transverse slice before and 1 h injection.

3.9) Histology examination of organs and tumor tissues with intravenous injection:

Mice were sacrificed after the multiple intravenous injections with NIR light mediated PDT treatment. The liver, lung, kidney, heart, spleen and tumor tissues were collected and fixed with 4% paraformaldehyde solutions. For histology analysis, all tumors and organs were processed routinely into paraffin and sectioned into 8 microns thick slices. Damaged cells in tumor region was performed using the terminal deoxyribonucleotide transferase-mediated nick-end labeling (TUNEL) assay using an *in situ* damaged cell detection kit (Roche, Basel, Switzerland) according to the manufacturer's instructions[19]. The extent of damaged was evaluated by counting the number of TUNEL-positive (brown-stained) cells, as shown in supplementary Fig. 24. The slices from various organs were also stained with hematoxylin & eosin (H&E) and examined by a digital optical microscope (BX 51, Olympus, USA), as shown in supplementary Fig. 25.

3.10) Bio-distribution studies:

Mice were sacrificed after the multiple intravenous injections with NIR light-mediated PDT treatment. The corresponding dissected organs (liver, lung, kidney, heart, spleen and tumor) were collected and weighted. The tissues samples were incubated in 70% nitric acid and digestion was allowed at 70 °C for several hours until the solution was clear[24,25]. After diluted with water to 2% acid solution, the solution was filtered with 0.22 μm filter and analyzed for yttrium (Y^{3+}) content by ICP-OES as shown in supplementary Fig. 26.

3.11) Statistical analysis:

Quantitative data are expressed as mean ± standard deviations (s.d.) unless specifically described. Significant differences can be determined using the Student's two-tailed t test where differences were considered significant ($p < 0.05$).

Supplementary References

1. Xie, X. J., *et al.* Mechanistic investigation of photon upconversion in Nd^{3+}-sensitized core-shell nanoparticles. *J. Am. Chem. Soc.* **135,** 12608-12611 (2013).
2. Zhong, Y., *et al.* Elimination of photon quenching by a transition layer to fabricate a quenching-shield sandwich structure for 800 nm excited upconversion luminescence of Nd^{3+}-sensitized nanoparticles. *Adv. Mater.* **26**, 2831-2837 (2014).
3. Wang, C., *et al.* Imaging-guided pH-sensitive photodynamic therapy using charge reversible upconversion nanoparticles under near-infrared light. *Adv. Funct. Mater.* **23,** 3077-3086 (2013).
4. Zhao, L., *et al.* Stem cell labeling using polyethylenimine conjugated (α-$NaYbF_4$:Tm^{3+})/CaF_2 upconversion nanoparticles. *Theranostic*, **3**, 249-257 (2013).
5. Liang, G. L., Ren, H. J. & Rao, J. H. A biocompatible condensation reaction for controlled assembly of nanostructures in living cells. *Nat. Chem.*, **2,** 54-60 (2010).
6. Qian, H. S., Chen, G., Ho, P. C., Mathendran, R. & Zhang Y. Mesoporous silica-coated up-conversion fluorescent nanoparticles for photodynamic therapy. *Small*. **20**, 2285-229 (2009).
7. Dragulescu-Andrasi, A., Kothapalli, S. R., Tikhomirov, G. A., Rao, J. H., & Gambhir, S. S. Activatable oligomerizable imaging agents for photoacoustic imaging of furin-like activity in living subjects. *J. Am. Chem. Soc.* **135,** 11015-11022 (2013).
8. Sigma-Aldrich, Antipain dihydrochloride, https://www.sigmaaldrich.com/content/dam/sigma-aldrich/docs/Sigma/Datasheet/6/a6191dat.pdf (2015).
9. Chen, Z., Wang, Z., Chen, J., Wang, S. et al. Sensitive and selective detection of glutathione based on resonance light scattering using sensitive gold nanoparticles as colorimetric probes. *Analyst* **137**, 3132-3137 (2012).
10. Cusabio, Human cathepsin B (CTSB) ELISA kit, https://www.cusabio.com/ELISA-Kit/Human-cathepsin-B-CTSB-ELISA-kit-115354.html (2015).
11. Thermo Fisher Scientific, Thermo scientific pierce protein assay technical handbook, https://www.thermofisher.com/content/dam/LifeTech/Images/integration/1602063_PAssayHB_122910.pdf (2015).
12. Cusabio, Human Folate receptor alpha (FOLR1) ELISA kit, https://www.cusabio.com/ELISA-Kit/Human-Folate-receptor-alphaFOLR1-ELISA-kit-78958.html (2015).
13. Huang, P., *et al.* Light-triggered theranostics based on photosensitizer-conjugated carbon dots for simultaneous enhanced-fluorescence imaging and photodynamic therapy. *Adv. Mater.* **24,** 5104-5110 (2012).
14. Sigma-Aldrich, *In vitro* toxicology assay kit (resazurin based), https://www.sigmaaldrich.com/content/dam/sigma-aldrich/docs/Sigma/Bulletin/tox8bul.pdf (2015).
15. Chris, J. H. H., *et al.* Multifunctional photosensitizer-based contrast agents for photoacoustic imaging. *Scientific Reports* **4,** 5342 (2014).
16. Sheng, Y, Liao, L. D., Thakor. N. & Tan, M. C. Rare-earth doped particles as dual-modality contrast agent for minimally-invasive luminescence and dual-wavelength photoacoustic imaging. *Scientific Reports* **4**, 6562 (2014).
17. Sigma-Aldrich, Mayer's hematoxylin solution, https://www.sigmaaldrich.com/content/dam/sigma-aldrich/docs/Sigma/General_Information/1/mhs.pdf (2015).
18. Rong, P., *et al.* Photosensitizer loaded nano-graphene for multimodality imaging guided tumor photodynamic therapy. *Theranostics* **4**, 229-239 (2014).
19. Invitrogen, APO-BrdU™ TUNEL Assay Kit, https://tools.thermofisher.com/content/sfs/manuals/mp23210.pdf (2015).
20. Li, J. C., *et al.* Polyethyleneimine-mediated synthesis of folic acid-targeted iron oxide nanoparticles for *in vivo* tumor MR imaging. *Biomaterials* **34,** 8382-8392 (2013).
21. Ye, W. L., *et al.* Cellular uptake and antitumor activity of DOX-hyd-PEG-FA nanoparticles. *PLoS One* **9,** e97358 (2014).
22. Cui, S. S., *et al. In vivo* targeted deep-tissue photodynamic therapy based on near-infrared light triggered upconversion nanoconstruct. *ACS Nano* **7**, 676-688 (2013).
23. Wang, C., Tao, H., Cheng, L. & Liu, Z. Near-infrared light induced *in vivo* photodynamic therapy of cancer based

on upconversion nanoparticles. *Biomaterials* **32**, 6145-6154 (2011).

24. Crayton, S. H., Elias, D. R., Zaki, A. Al, Cheng, Z. & Tsourkas, A. ICP-MS analysis of lanthanide-doped nanoparticles as a non-radiative, multiplex approach to quantify biodistribution and blood clearance. *Biomaterials* **33**, 1509-1519 (2012).
25. Cheng, L., *et al.* Multifunctional nanoparticles for upconversion luminescence/MR multimodal imaging and magnetically targeted photothermal therapy. *Biomaterials* **33**, 2215-2222 (2012).
